# Home surveillance system based on LoRa backscattering

**DOI:** 10.1038/s41598-025-96624-0

**Published:** 2025-04-08

**Authors:** Marc Lazaro, Antonio Lazaro, Ramon Villarino, David Girbau

**Affiliations:** Department of Electric, Electronic and Automatic Engineering, Rovira i Virgili University, 43007 Tarragona, Spain

**Keywords:** Backscattering, RFID, LoRa, Internet of Things, Battery free, Engineering, Electrical and electronic engineering

## Abstract

This work proposes a battery-free wireless burglar alarm system based on LoRa backscattering. The system consists of multiple wireless nodes that send an alert when its sensor is triggered. The primary goal is to significantly reduce the power consumption of these wireless nodes, eliminating the need for battery replacements and maintenance associated with commercial systems. The paper addresses several crucial aspects of the application, including the hardware design of the tag, which encompasses energy management up to the RF front-end. Additionally, this paper explores the wireless communication protocol by presenting a fully analog modulation approach to optimize power consumption and system responsiveness. This novel method leverages the intrinsic frequency detection function of commercial LoRa transceivers to determine the origin of backscattered LoRa packets, thereby enabling the integration of a large number of sensors within the same frequency channel, all using a single receiver. Finally, the paper provides a proof of concept validated through on-site measurements.

## Introduction

The rapid advancement of technology has transformed home automation from a luxury accessible only to a few into a widely available solution. Developments in the Internet of Things (IoT), reductions in manufacturing costs, and the standardization of protocols have enabled virtually anyone, even those without prior knowledge, to automate their homes. With the advent of artificial intelligence, it is now rare to find a household without a voice assistant, such as Amazon Alexa, Google Assistant, or Apple Siri. Automating home lighting is as simple as replacing old bulbs with smart ones and asking the voice assistant to synchronize them.

A subset of home automation focuses on home security. Unlike conventional security systems, which are typically installed and maintained by third-party companies, plug-and-play security systems have recently become commercially available. These systems are compatible with major voice assistants and common wireless technologies, gaining significant interest due to their ease of installation and the absence of monthly maintenance fees.

Unlike voice assistants or smart bulbs, which typically have access to a power source, low-cost security systems often integrate two types of sensors: magnetic sensors for door/window monitoring and passive infrared (PIR) sensors for motion detection. Given the installation points (windows, doors, elevated positions, etc.), wiring the sensors can be tedious, making battery power preferable. This leads to the objective of this research: designing a low-cost security system based on LoRa backscattering that reduces the power consumption of wireless nodes sufficiently to eliminate the need for batteries and, consequently, the periodic maintenance required by commercial systems.

Radiofrequency backscattering is the principle behind technologies like radar and radio frequency identification (RFID). The primary appeal of this technique is its inherently low power consumption. However, the double path loss inherent in this technique significantly reduces the signal-to-noise ratio, limiting its application to short-range communications for several decades. Additionally, the high cost of RFID readers and radar systems has restricted the adoption of backscattering within the IoT field. Nevertheless, recent proposals for low-cost transceiver applications based on backscattering have emerged, opening many possibilities in the IoT landscape^1^.

Recent advances in backscattering systems focus on addressing the main challenges associated with this technique: modulation^2^, range^3^, interoperability^4^, interference^5,6^, data rate^7^, and scalability^8^, among others. This work studies the design and characterization of a security system based on the backscattering of spread-spectrum LoRa signals, offering significant advantages over existing commercial systems. Additionally, various studies have examined the integration of low-power sensors, such as image sensors^9^, vibration sensors^10^, humidity sensors^11^, and temperature sensors^12^, alongside backscatter-based radiofrequency front-ends.

In addition to significantly reducing power consumption through backscattering techniques, we propose a novel, fully analog modulation system. This system leverages the frequency deviation detection functionality intrinsic to all LoRa transceivers to determine the origin of received packets. This approach enables the identification of a large number of tags using a single frequency channel and, consequently, a single receiver. Specifically, if each wireless sensor (tag) introduces a small, unique, and predefined frequency deviation, the receiver can utilize this information as an identifier. This modulation technique ensures superior system responsiveness compared to any digital modulation, making it particularly advantageous for surveillance applications.

## State-of-the-art on LoRa backscattering

Contributions in the field of LoRa backscattering have garnered significant interest over the past decade due to their inherent potential for low-power applications. This section aims to provide a detailed review of the most significant contributions in LoRa backscattering, supporting the work presented in the following paper.

A large number of contributions have focused on both enhancing LoRa backscattering technology and bridging the gap between research and the commercial sector by proposing new applications or analyzing the benefits of integrating this technology into existing systems. We previously proposed the design of a low-resolution localization system^13^ and a deep implanted medical device^14^, both systems integrating LoRa backscattering to implement the wireless communication. Jiang et al propose a technology to sense the cyclist conditions by attaching a backscattering tag that leverages the LoRa communications already present in the public bicycle sharing systems^15^. Katanbaf et al propose the design and implementation of a full duplex LoRa backscatter reader and propose its application for being integrated in smartphones, to read smart lenses via backscattering, and in drones, from smart agriculture purposes. Zhang et al. propose a LoRa backscatter-assisted state estimator for micro aerial vehicles, enabling efficient online initialization and robust state estimation in challenging environments^16^. Hou et al. present LoBaCa, a super-resolution LoRa backscatter localization system that enables precise, low-cost tracking of tags using advanced signal processing techniques, achieving an error of only 5 cm and 71 cm when the tag is 5 m and 40 m away, respectively^17^. Liu et al. present LoMu, a long-range multi-target backscatter sensing system that enables precise and low-cost tracking of multiple tags simultaneously. Compared to articles^15^ and^16^, LoMu improves localization accuracy, extends operational range, and enhances scalability by leveraging advanced signal processing and interference mitigation techniques^18^.

On the other hand, several publications have focused on enhancing key aspects of LoRa backscattering technology, including power consumption, communication range, interoperability, and transmission speed, among others. Some of the most notable works in this area are mentioned next. In 2017, Talla et al present the first wide-area backscatter communication system based on LoRa technology, achieving distances up to 2.8 km while consuming only 9.25 $\upmu {\rm W}$^19^. This work represents a milestone in the advancement of backscattering communications. In 2018, Peng et al. present PLoRa, a passive long-range ambient LoRa backscatter system that enables battery-free IoT devices to communicate over distances up to 1.1 km by leveraging ambient LoRa transmissions as excitation signals, introducing a novel blind chirp modulation algorithm and an energy-efficient hardware design^20^. In 2020, Guo et al. present Aloba, an ambient LoRa backscatter system that employs the classic ON-OFF Keying modulation within the band of the carrier modulation, thus improving the spectral efficiency and throughput compared to previous works^21^. This contribution states data-rates up to 199.4 kbps. In 2020, Li et al. present XORLoRa, a LoRa backscatter communication system that enables data transmission using only commodity LoRa devices by leveraging frequency shifting and a novel encoding strategy, achieving a communication range of 30 m indoors and 500 m in open space with a peak throughput of 1.6 kbps^22^. In 2021, Jiang et al. present P2LoRa, the first LoRa backscatter system with parallel decoding, enabling long-range communication while supporting up to 101 simultaneous backscatter transmissions by leveraging frequency shifting and interference cancellation techniques^3^. In 2021, Katanbaf et al. present a Full-Duplex LoRa Backscatter reader, leveraging low-cost passive components and adaptive impedance tuning to achieve 78 dB self-interference cancellation, simplifying deployment and enabling long-range, low-power backscatter communication^23^. In 2021, Sheikh et al. present an ultra-low-power wide-range backscatter communication system using cellular-generated carriers, evaluating its feasibility for smart city applications through extensive simulations and demonstrating its potential for large-scale IoT deployments^8^. In 2022, Guo et al. present Saiyan, a low-power demodulator for long-range LoRa backscatter systems that enables backscatter tags to demodulate feedback signals from a remote access point, improving packet retransmission efficiency, channel hopping, and data rate adaptation^24^. In 2022, Peng et al. present Pacim, an ambient LoRa backscatter system that introduces chirp interval modulation to encode multiple bits per symbol, along with a twin-chirp cancellation method and a fine-grained detection algorithm, ultimately achieving 8.6 times the throughput gain stated in literature^25^. In 2022, Xiao et al. present BackLoRa, a backscatter-assisted LoRa transmission system that enhances collision resilience by leveraging backscatter tags to create multipath propagation features, reducing the symbol error rate from 65.3 to 5.5% and considerably improving throughput in low-SNR conditions^26^. In 2023, Lin et al. analyze the temporal, spectral, and error performance of LoRa backscatter communications, proposing a signal model that accounts for the limited number of loads in the tag and deriving closed-form expressions for power spectrum and symbol error rate under different channel conditions^27^. In 2023, Ren et al. present Prism, a high-throughput LoRa backscatter system that enables concurrent transmissions by leveraging non-linear chirps, allowing up to 700 tags to transmit simultaneously with considerably increase in transmission concurrency compared to state-of-the-art approaches^28^. Recently, in 2024, Tang et al. present a prototype implementation and experimental evaluation of a LoRa-backscatter communication system with RF energy harvesting and low-power management, introducing an ultra-low-power LoRa tag that operates with as little as -19 dBm of harvested RF energy and achieves a communication range of 445 m^29^. Table 1 compares and summarizes the most relevant contributions in the field of LoRa Backscatter.

**Table 1:** Comparison of the most relevant LoRa backscattering contributions.

Author	Year	Carrier	Receiver	Consumption	Throughput	Tag-Rx
Talla et al^19^	2017	Frequency tone (905 MHz)	Commodity (LoRa: SX1276)	9.25 $\upmu {\rm W}$ (IC)	18 bps–37.5 kbps	237.5 m (2.8 km, Tx-Tag: 5 m)
Varshney et al^30^	2017	Frequency Tone (CC2420) (0.86-2.4 GHz)	Custom (CC1310-868) (CC2500-2.4)	70 $\upmu W$ (868) 650 $\upmu W$ (2.4)	2.9/197 kbps (868/2.4)	3.4 km (868) 225 m (2.4)
Peng et al^20^	2018	LoRa Gateway (900 MHz)	Commodity (LoRa)	220 $\upmu W$	6.25 kbps (SF7) 97 bps (SF12)	300 m (SF7) 800 m (SF12)
Guo et al^21^	2020	LoRa (900 MHz)	Custom (SDR: USRP)	300 $\upmu W$	9.9–121.4 kbps	71.4–225.6 m
Katanbaf et al^23^	2021	Frequency tone (ADF4351) (900 MHz)	Custom (Tx-Rx co-loc) (ADF+SX1276)	(Receiver) 112 mW(4 dBm) 3.04 W (30 dBm)	366 bps–3.6 kbps	91.44 m
Jiang et al^3^	2021	LoRa (433 MHz)	Custom (SDR:USRP N210)	320 $\upmu {\rm W}$	120 bps	2.2 km
Guo et al^31^	2021	LoRa (900 MHz)	Custom (SDR:USRP N210)	Receiver: 0.3 mW	39.5–199.4 kbps	50-200 m
Guo et al^24^	2022	LoRa (469.65 MHz)	LoRa Receiver (SX1278)	Receiver: 93.2 (IC)	19.6 kbps	180 m
Ren et al^28^	2023	Linear chirp	Custom SDR: USRP	695 $\upmu {\rm W}$	68.36 kbps	600 m (Tx-Tag: 5m)
Tang et al^29^	2024	Frequency tone (433 MHz)	Custom (COTS design)	Tag Rx: 1.74$\upmu {\rm W}$ Tag Tx: 251 $\upmu {\rm W}$	18 $\sim$ 38 kbps	445 m

## System architecture

**Fig. 1:**
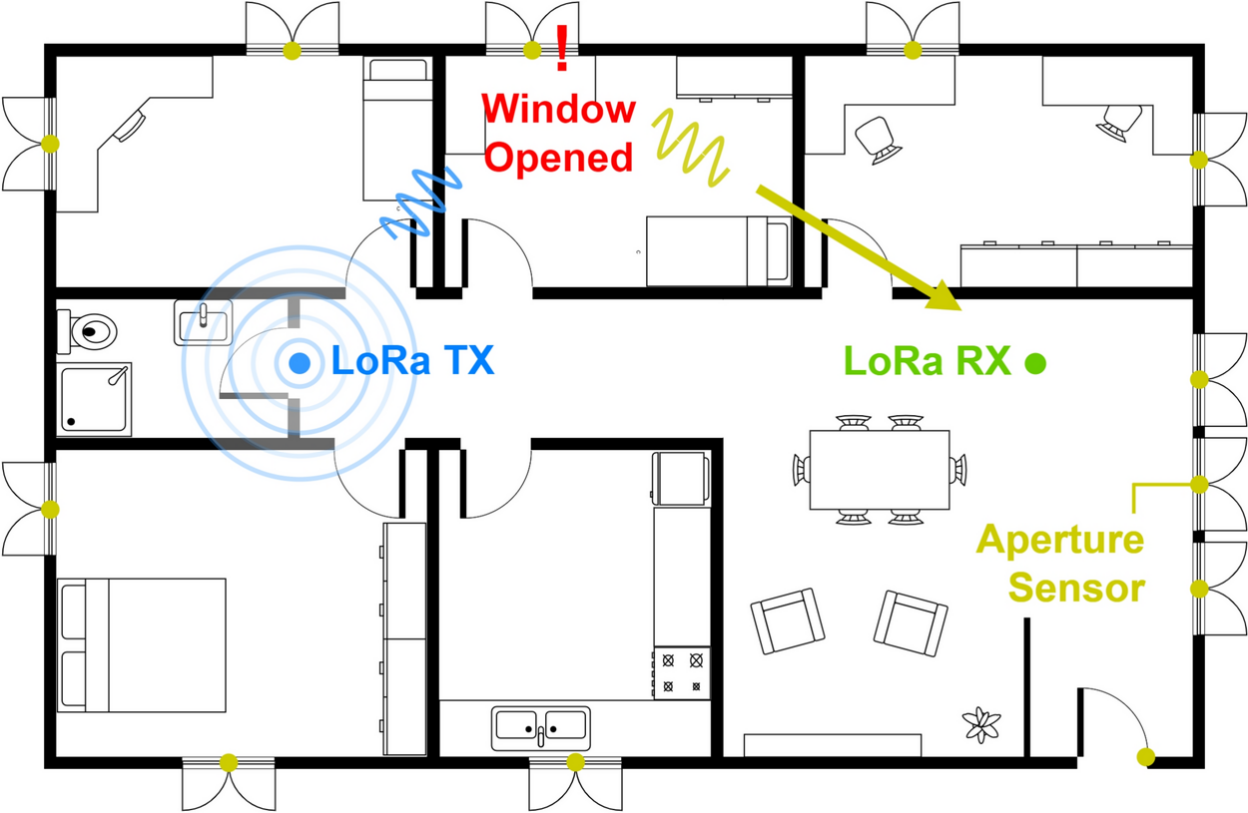
Deployment diagram of the proposed system.

Figure 1 provides an overview of the proposed system’s operation. The setup includes a LoRa transmitter, a LoRa receiver, and wireless nodes installed at key monitoring points, such as entrance doors and windows. The transmitter periodically sends LoRa packets in the 433 MHz ISM band. When a wireless node is activated (the magnetic sensor is triggered), it backscatters the incoming LoRa packets to adjacent channels (433 ± 300 kHz), where the receiver is tuned to detect the signal. The receiver can detect the transmission on either of the two sidebands. Thus, this system provides greater robustness against narrowband jamming attacks. Additionally, for wideband jammers, increasing the modulation frequency of the tag ($\Delta f$) requires the jamming device to increase its bandwidth by a factor of two ($2 \times \Delta f$) in order to interfere with the signal.

The proposed backscattering system employs a bistatic architecture, where the transmitter and receiver are positioned separately. Compared to monostatic configurations, bistatic systems offer several advantages. Path losses are generally lower, as they avoid round-trip propagation. Additionally, separating the transmitter and receiver reduces system complexity and minimizes interference between the two, addressing the ’doubly near-far’ problem^32–34^. This architecture also provides greater flexibility by allowing independent placement of the transmitter and receiver. Optimizing their locations -or increasing the number of transceivers- can help compensate for environment factors such as multipath interference or signal attenuation, significantly improving the system’s range and reliability^35^.

### Carrier modulation

**Fig. 2:**
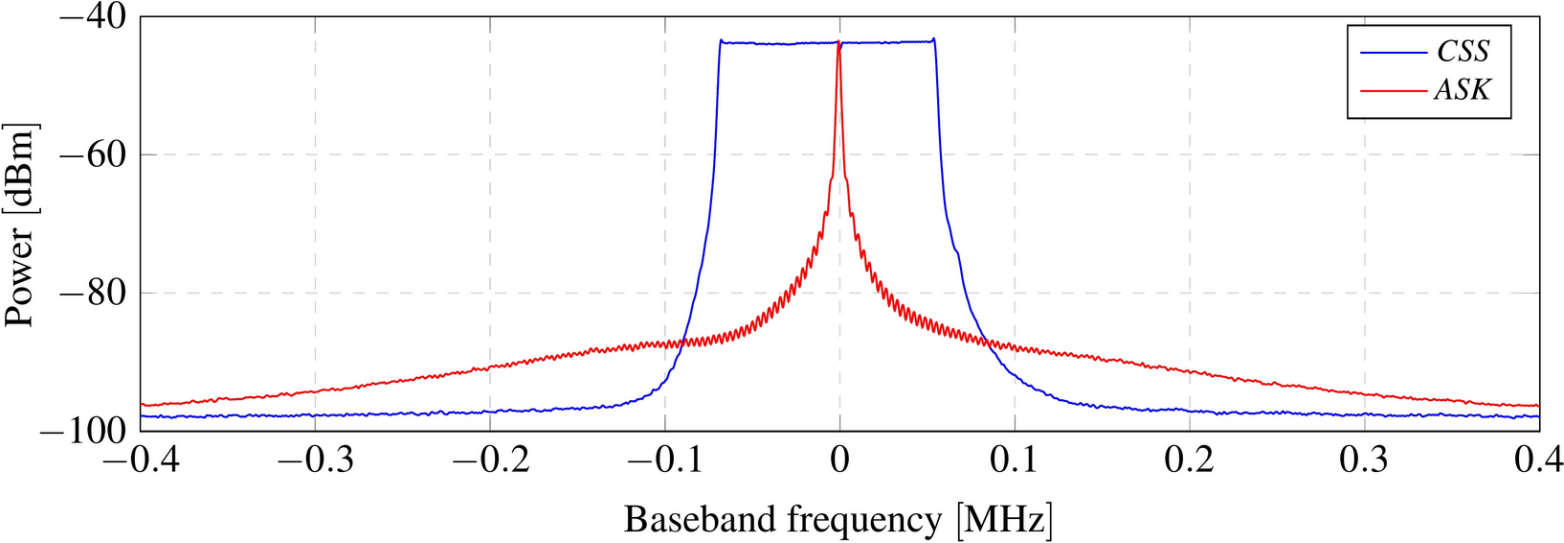
Comparison of a commercial sensor transmission (amplitude shift keying, ASK) with the proposed system’s carrier modulation (LoRa) at 433 MHz.

Home surveillance systems can operate across various frequency bands, ranging from 300 MHz to 2.4 GHz. Despite the existence of companies utilizing private frequency bands, modulations, and protocols, there is a prevailing trend towards standardization at 433 MHz due to its balanced trade-off between attenuation and throughput^36,37^. Typically, commercial sensors employ well-established modulation techniques such as amplitude shift keying (ASK) and on-off keying (OOK). These methods maintain a low transceiver complexity, resulting in reduced costs. However, it is important to note that amplitude modulation is susceptible to noise and interference, which may seem counterintuitive given its widespread use in wireless security systems. Countering jamming attacks is a difficult task. While any known wireless transmission can be interfered with, there are modulations and techniques, such as band hopping, reception confirmation, and spread spectrum modulations, designed to make this task more challenging. This work proposes using LoRa as the carrier modulation, bringing several advantages to the already-in-use ASK/OOK modulation. LoRa is a proprietary wireless technology based on the chirp spread spectrum (CSS) technique^38^. CSS signals are inherently resistant to multipath and narrowband interference, unlike amplitude modulation. Additionally, this technique provides very long-range wireless communications, up to several kilometers, with a low power consumption, making it ideal for this application. Figure 2 shows the spectrum of an amplitude shift keying (ASK) signal transmitted by a commercial sensor, overlapped with the CSS signal employed in this work. While the robustness to interference makes LoRa ideal for this application, its long-range capability is particularly advantageous for compensating for the double path loss associated with the backscattering technique. The CSS demodulation process involves multiplying the incoming up-chirp by a down-chirp with identical properties -bandwidth (BW) and spreading factor (SF)- resulting in a constant frequency signal with significant processing gain, as mathematically demonstrated in^39^. LoRa technology stipulates various predefined configurations to balance the trade-off between speed and coverage. LoRa transceivers allow for the configuration of multiple parameters of the CSS modulation, such as bandwidth, ranging from 7.8 to 500 kHz, and spreading factors, from 6 to 12. Additionally, parameters such as coding rate, sync word, preamble length, and cyclic redundancy checksum (CRC) can also be configured. Based on these settings, the airtime of the LoRa transmission can be calculated. For this application, the CSS signal is configured to maintain an equilibrium between airtime and coverage, utilizing a bandwidth of 125 kHz, a spreading factor of 12, a 4/5 coding rate, a preamble of 8 chirps, and no CRC. With these settings, the airtime of a LoRa packet is 663.55 ms.

### Backscattering principle

Backscattering techniques have been extensively studied in the literature^1,40,41^. The principle behind consists in reflecting the impinging electromagnetic fields instead of being natively generated by the radio. This technique, as previously mentioned, is the basis of technologies like radar and RFID. Backscattering can be accomplished by modulating the reflection coefficient ($\Gamma$) at the antenna terminals by changing the loading impedance. A simple but effective and widely used way is switching between a short circuit and an open circuit. The amplitude and phase of the backscattered signal depend on the resistance and reactance of the antenna load, while the frequency depends on the load switching frequency. This switching process is identical to the most basic implementation of a mixer. Therefore, assuming that the backscattering tag is illuminated by a LoRa signal *L*(*t*), when the tag is modulated by the signal *N*(*t*), the backscattered signal *B*(*t*) can be mathematically modeled as the multiplication of these two signals as follows:

1 $$\begin{aligned} \begin{aligned} B(t)&= L(t) \cdot N(t) = A \cdot cos(\omega _Lt + \frac{BW}{2T}t^2) \cdot sgn(cos(\omega _T t))\\&= \frac{2A}{\pi } \sum _{n=1,3..}^\infty \frac{1}{n} [cos((\omega _L + \frac{BW}{2T}t - n\omega _T)t) - cos((\omega _L + \frac{BW}{2T}t + n\omega _T)t)] \end{aligned} \end{aligned}$$

where, *L*(*t*) is mathematically defined by the chirp function, with an initial frequency $\omega _L$, amplitude *A*, bandwidth *BW*, and period *T*, and *N*(*t*) is modeled as a square wave with a frequency equal to $\omega _T$.

### Tag identification

Wireless security systems do not require a high data rate but necessitate a fast response, as they must alert the central unit when the sensor is triggered by sending a short packet. The objective of this work is to significantly reduce the power consumption of the wireless sensors to make them battery-free. Based on this premise, the proposed system implements a completely analog modulation, thereby reducing the active time compared to a digital modulation and consequently lowering power consumption. For the implementation of this modulation, commercial LoRa transceivers, without any modifications, are used, making the system more cost-effective and efficient compared to applications that use an SDR as a transceiver.

Wireless nodes backscatter the incoming LoRa packets to the sideband when their sensor is triggered. To distinguish packets from each wireless node, each node can be configured to modulate at a different frequency channel (e.g., Node 1: ±300 kHz, Node 2: ±600 kHz, etc.), thereby avoiding packet overlap. However, this technique considerably limits the number of nodes that the system can manage, as it requires a receiver to listen on each channel. This, in turn, increases both the cost and the amount of spectrum used, making it impractical.

**Fig. 3:**
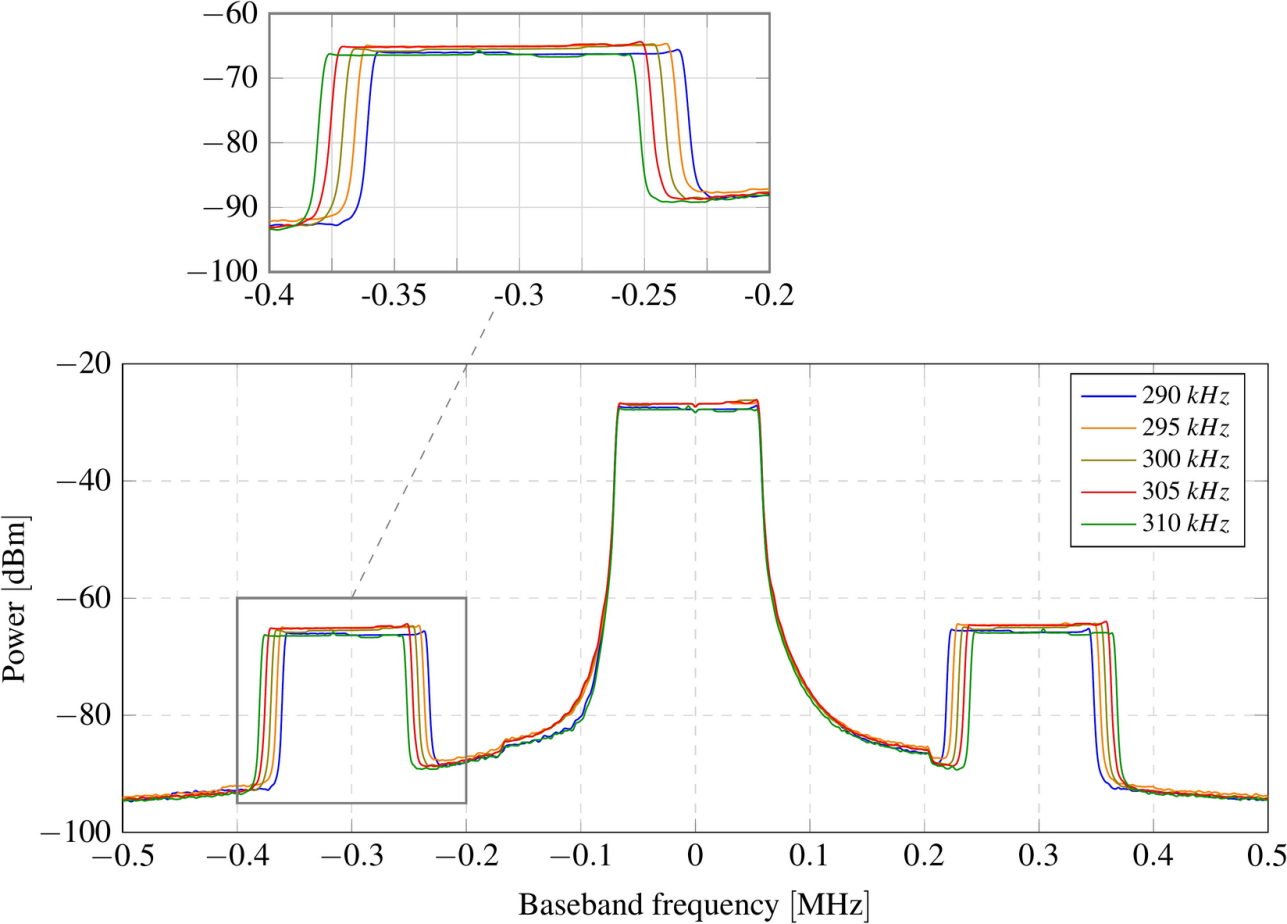
Artificial frequency deviation introduced by each tag in the sideband channel. Carrier modulation: LoRa at 433 MHz; tag modulation frequencies: 290 kHz, 295 kHz, 300 kHz, 305 kHz, and 310 kHz.

LoRa transceivers from Semtech (SX12xx, LR1121, and LLCC68) natively include a frequency correction function that compensates for oscillator drift. The receiver correlates the incoming signal with the previously known preamble of the packet (a train of up-chirps), synchronizing both signals for the demodulation process. The user can read the value of this deviation in hertz from the memory register. Leveraging this function, which is inherent in all LoRa receivers, all wireless nodes can backscatter packets on the same sideband channel, optimizing spectrum usage. Each node introduces an artificial frequency deviation that the receiver compensates for during the correlation process. By checking the frequency deviation, it can be determined which node sent the packet. Figure 3 shows the measured spectrum of the backscattered LoRa packets for five different nodes. The frequency offset introduced by each tag has been spaced to be visually perceptible; however, in practice, it can be set to be much lower.

LoRa technology (CSS modulation), compared to other types of modulation such as frequency shift keying (FSK) or orthogonal frequency division multiplexing (OFDM), does not require precise oscillators, as the demodulation process can compensate for frequency deviation. For this reason, the oscillators in some LoRa transceivers exhibit considerable frequency drift. While this is not typically a problem under normal conditions, it poses an issue in this application, where we aim to identify wireless nodes by introducing a fixed frequency deviation. Semtech’s datasheets indicate that LoRa transceivers can operate with a frequency deviation of less than or equal to ±25% of the bandwidth^42^. For instance, with a bandwidth of 62.5 kHz, the LoRa receiver would accept a frequency deviation of ±15.625 kHz.

**Fig. 4:**
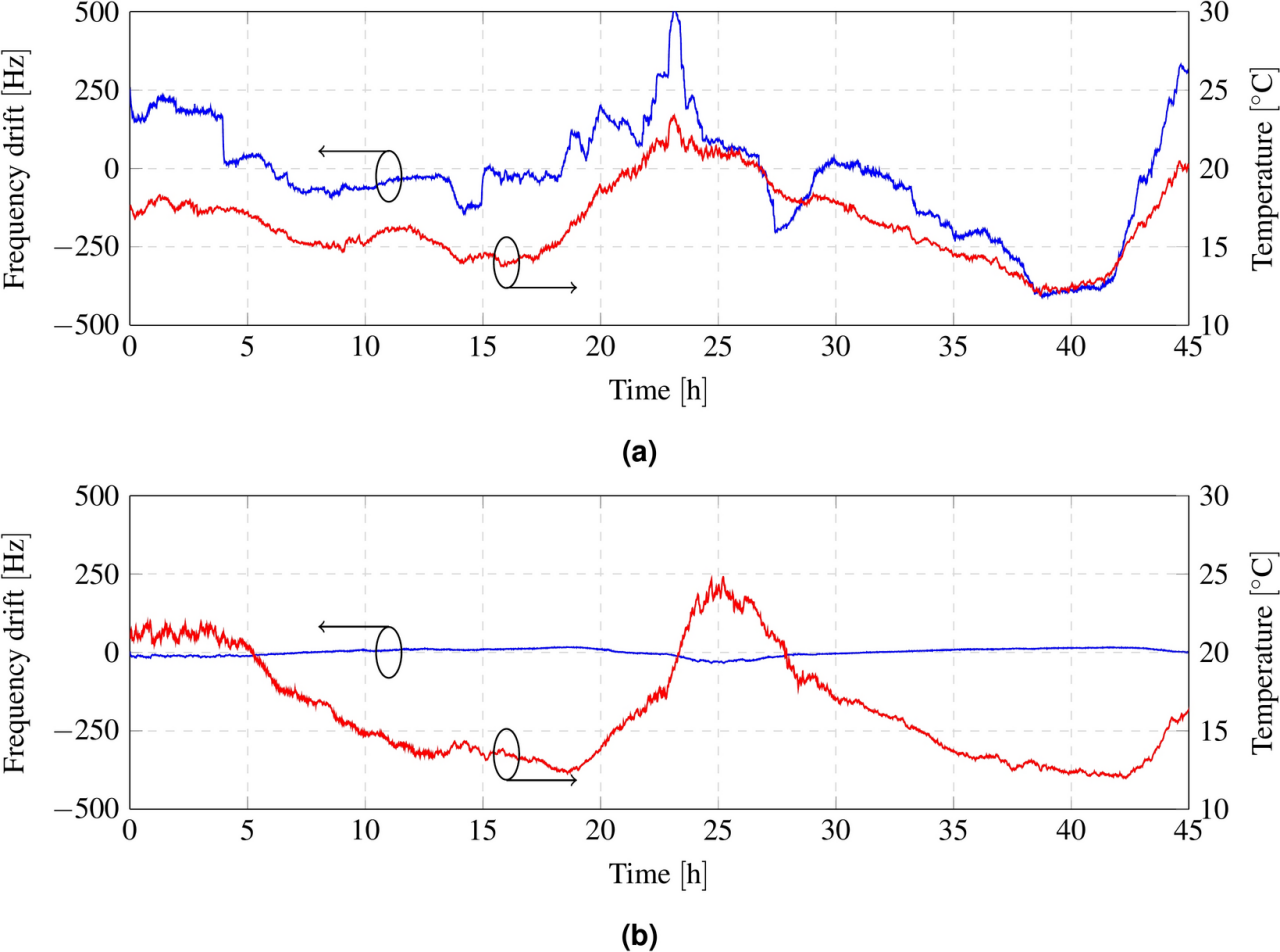
Comparison of the measured oscillator frequency deviation (blue) as a function of temperature (red) for the SX127X family (**a**) and SX126X family (**b**) of transceivers.

Many commercial LoRa modules integrate the SX127X transceivers, which exhibit considerable drift in oscillator frequency. Semtech released the SX126x transceivers as an upgraded version, incorporating a temperature-compensated oscillator (TCXO) that addresses the issues of its predecessor. Figure 4 shows the measured frequency deviation as a function of temperature for both transceivers. In this work, an SX1262 transceiver is used due to its oscillator frequency stability; however, the frequency of SX1276 transceivers can alternatively be compensated manually by performing a regression analysis of the frequency drift as a function of temperature. This approach requires integrating a temperature sensor with the transceiver.

Observing Fig. 4, it can be seen that the frequency fluctuates by more than 977 Hz for the SX1276 transceiver (Fig. 4a), while it fluctuates by only 52 Hz for the SX1262 (Fig. 4b). As mentioned in the carrier modulation subsection, LoRa transceivers support various configurations to balance the trade-off between communication range and throughput. For this application, a standard configuration is chosen, with a bandwidth (BW) of 125 kHz and a spreading factor (SF) of 12. However, depending on the environment, the BW can be reduced to increase communication range, or the SF can be decreased to enhance throughput, and vice versa. With this configuration, the receiver supports a frequency deviation of the demodulated packet of ±31.25 kHz. Considering the frequency deviation measurement of ±26 Hz and adding an additional margin of 74 Hz, the system is capable of supporting a maximum of 625 sensors.

### Tag implementation

**Fig. 5:**
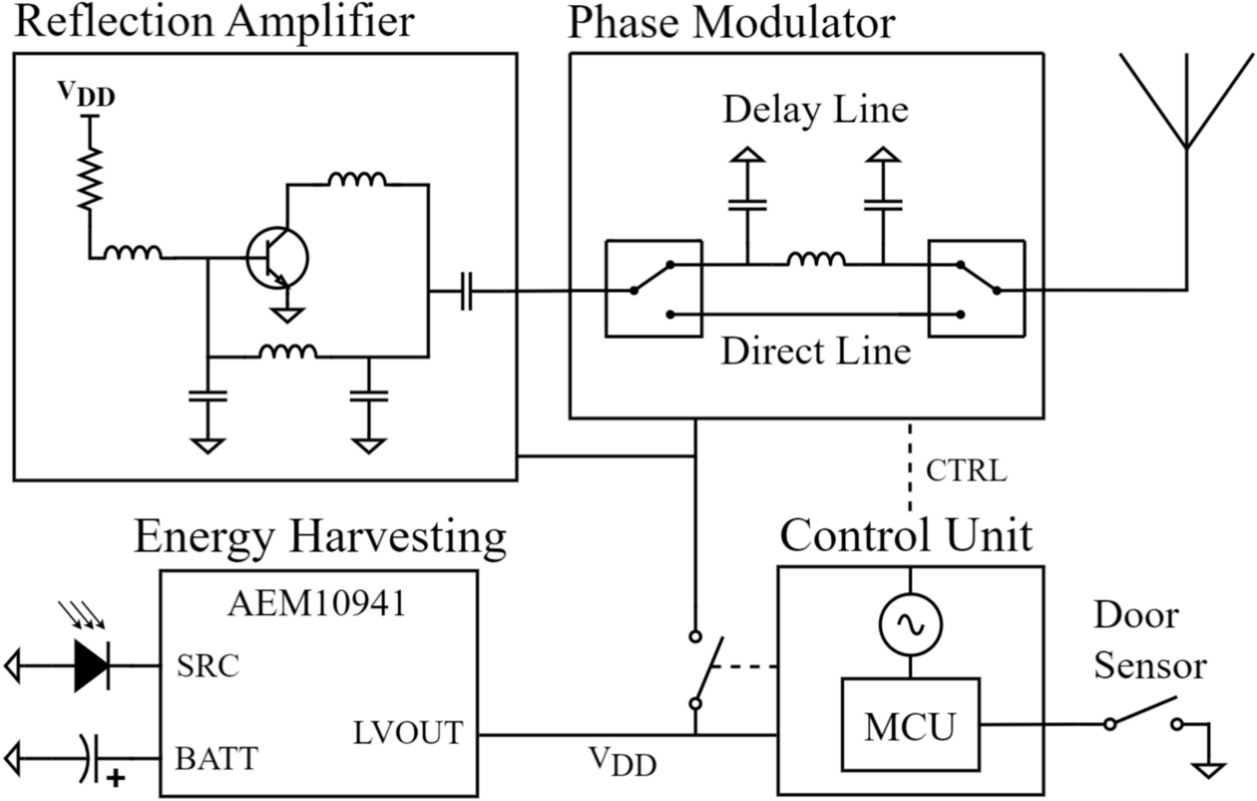
Block diagram of the wireless node (tag).

This section presents the design of the wireless backscattering node. The backscattering tag consists of four distinct parts, as shown in Fig. 5: the reflection amplifier, phase modulator, control unit, and energy harvesting/management unit. Each part is implemented separately for proof of concept, enabling the individual characterization of each part. The two blocks constituting the radio frequency front-end are fabricated on 32 mils Rogers 4003C substrate.

#### Reflection amplifier

The reflection amplifier allows for increasing the differential radar cross-section by generating a reflection coefficient greater than one. This type of amplifier is characterized by having a single input and output port. In the literature, dual-antenna backscattering tags using dual-port amplifiers have been proposed^43^. This topology works well with highly directional antennas and within the SHF frequency range, where attenuation is higher. However, at frequencies below 1 GHz, when paired with isotropic antennas, this topology tends to oscillate due to parasitic coupling between the antennas.

The reflection amplifier can be designed with various active devices such as diodes or transistors (IMPATT, Gunn, Tunnel, BJT, FET, etc.). Tunnel diodes have gained considerable interest recently due to their outstanding efficiency. However, with advances in semiconductor technology, the use of tunnel diodes has significantly declined, rendering them almost obsolete due to their complex fabrication. Currently, there are few manufacturers supplying this type of device, and consequently, they are considerably more expensive and difficult to obtain than transistors^44^.

**Fig. 6:**
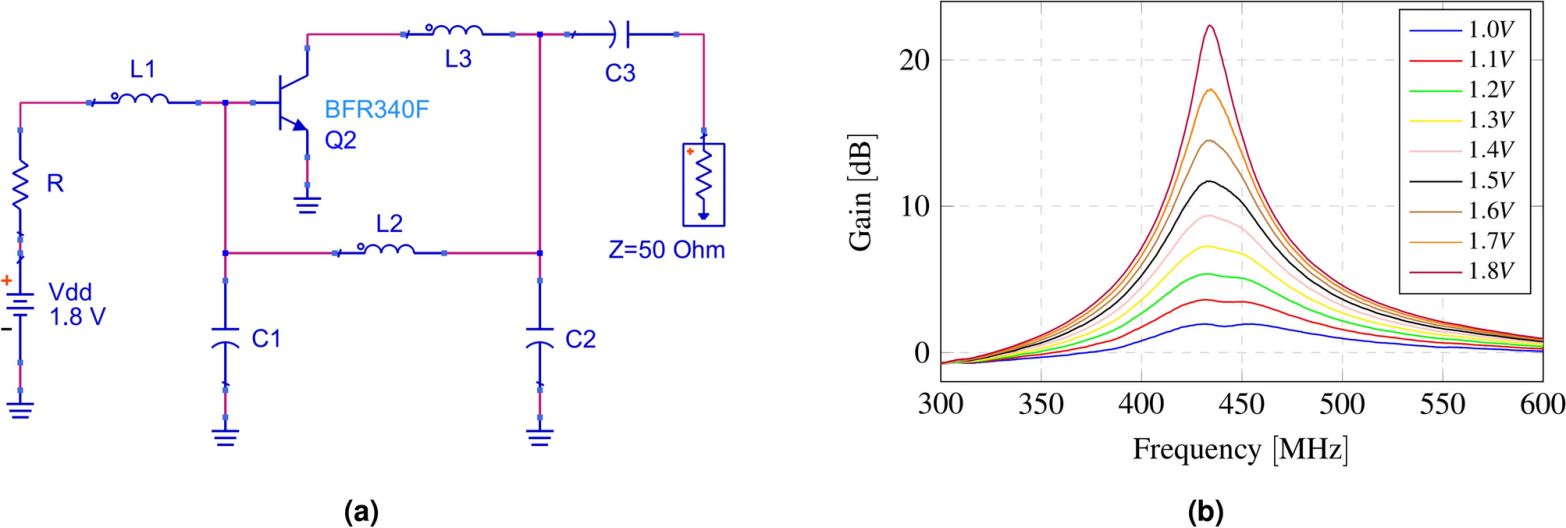
(**a**) Schematic of the reflection amplifier. Topology: common-emitter with a pi-network feedback loop. $R = 300$ $\Omega$, $L1 = 260$ nH, $L2 = 15$ nH, $L3 = 82$ nH, $C1 = 15$ pF, $C2 = 15$ pF, $C3 = 33$ pF; (**b**) Reflection amplifier gain as a function of bias voltage.

In this work, the reflection amplifier is implemented using a BFR340F bipolar transistor from Infineon Technologies. The reflection amplifier operates on the same principle as any oscillator, generating a negative resistance at the input/output port. Traditional oscillator topologies, such as Hartley, Clapp, or Colpitts, can be employed to implement a reflection amplifier. In this case, a common-emitter topology with a negative feedback branch is used. The feedback branch is designed as a pi network, tuned as a bandpass filter at 433 MHz. Figure 6a shows the schematic of the reflection amplifier.

**Fig. 7:**
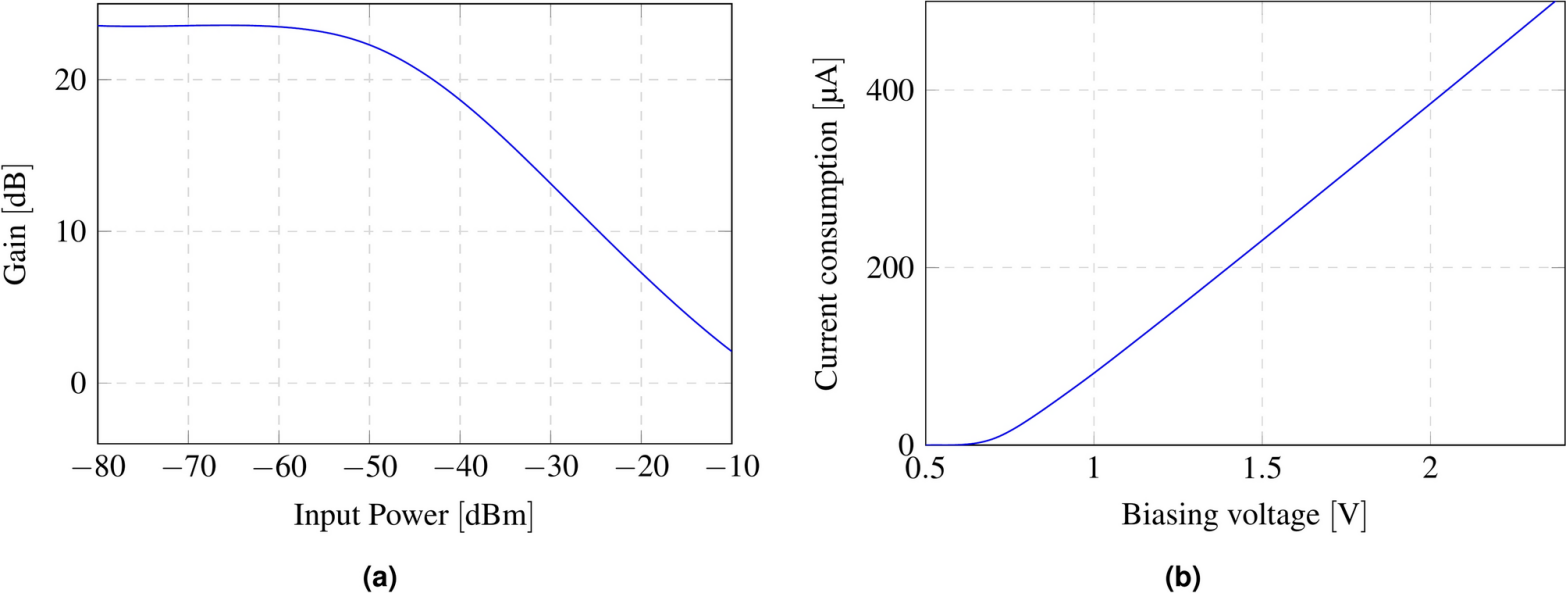
(**a**) Reflection amplifier gain as a function of input power; (**b**) current consumption of the reflection amplifier as a function of bias voltage.

The reflection amplifier is characterized with the network analyzer configured with an output power of − 50 dBm. Figure 6b presents its gain as a function of the transistor’s biasing voltage. Figure 7a shows the amplifier gain as a function of input power. It can be observed how it decreases as the input power increases, as expected due to the amplifier’s gain compression. Figure 7b shows the current consumption of the amplifier as a function of the biasing voltage. The current consumption and the gain of the reflection amplifier when polarized at 1.8 V are 322.9 $\upmu {\rm A}$ and 22.3 dB, respectively.

#### Phase modulator

**Fig. 8:**
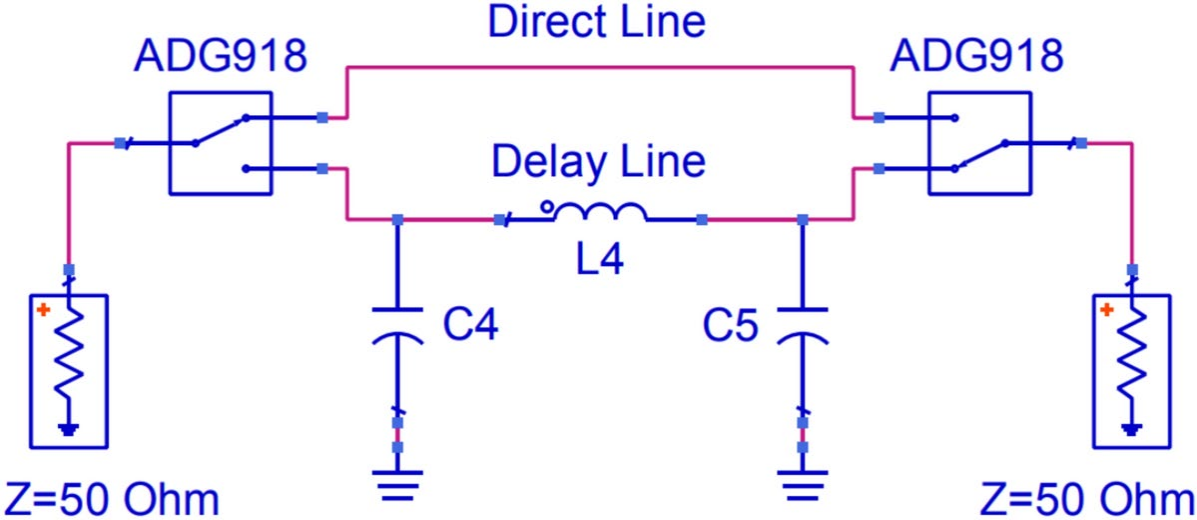
Schematic of the phase modulator. $L4 = 18$ nH, $C4 = 6.8$ pF, $C5 = 6.8$ pF.

Maximizing the differential radar cross-section involves generating two reflection coefficients that are 180 degrees out of phase with each other^45^. To achieve this, a phase modulator is integrated between the antenna and the reflection amplifier. The phase modulator is implemented using two RF switches (ADG918), which switch between a direct line (no delay) and a delay line specifically designed to introduce a 90-degree phase shift at 433 MHz. As the signal passes through the modulator twice, it becomes 180 degrees out of phase compared to the direct line. The delay line can be constructed with discrete components or microstrip technology. The first option introduces higher losses, especially at high frequencies, and is more complicated to implement due to the tolerance of discrete components. The second option introduces fewer losses and has lower manufacturing errors; however, at 433 MHz, a 90-degree phase shift line results in a significantly larger design. For this reason, discrete components have been selected. Figure 8 illustrates the schematic of the implemented phase modulator.

**Fig. 9:**
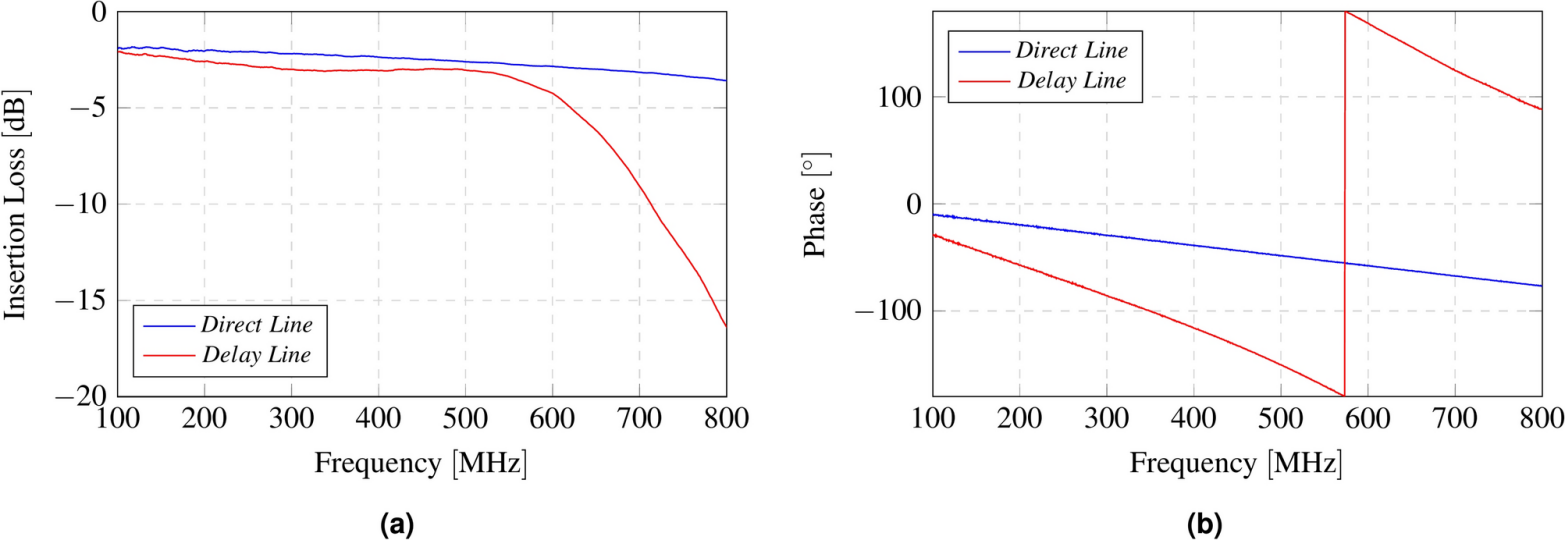
Characterization of the phase modulator for both states: direct line and delay line. Insertion Loss (**a**) and phase (**b**) as a function of frequency.

Figure 9 shows the characterization of the phase modulator. A phase shift of 84.28 degrees between the two states can be observed, quite close to 90 degrees, with insertion loss of 2.43 dB and 3 dB for the direct line and delay line, respectively, at 433 MHz. It can be observed that the insertion loss for the delay line with the pi network increases significantly with frequency, making this topology not appropriate for designs at higher frequencies.

#### Energy harvesting

There are several sources for energy harvesting (solar, thermal, kinetic, electromagnetic, etc.), but not all of them are suitable for any application. In the field of wireless sensor networks (WSN), the most common source is solar energy^46^. Although this technique is not suitable for dark environments, such as garages, the continuous improvement in solar panel efficiency and integrated energy management chips has made it possible to harvest energy even in indoor environments without natural light. Harvesting energy through electromagnetic fields is also a widely used technique in the field of RFID and backscattering tags. However, this type of harvesting involves absorbing part of the carrier energy instead of reflecting it, which reduces the differential radar cross-section of the tag and consequently the communication range. To avoid this disadvantage, two antennas can be integrated: one for the backscattering front-end and another for the energy harvesting system. However, the operating range of this energy harvesting system remains quite restricted for some applications, ranging from 7 to 15 m at best^46^.

For the tag design, a light-based energy harvesting system is chosen, as this system does not limit the communication range of the tags. For proof of concept, an AEM10941 energy harvesting and management integrated circuit is utilized. This chip allows energy harvesting from an input power as low as 3 $\upmu {\rm W}$ and a voltage of 380 mV. It also integrates a low-dropout (LDO) regulator with 1.2 V and 1.8 V outputs, making it ideal for powering low-power consumption ICs.

**Fig. 10:**
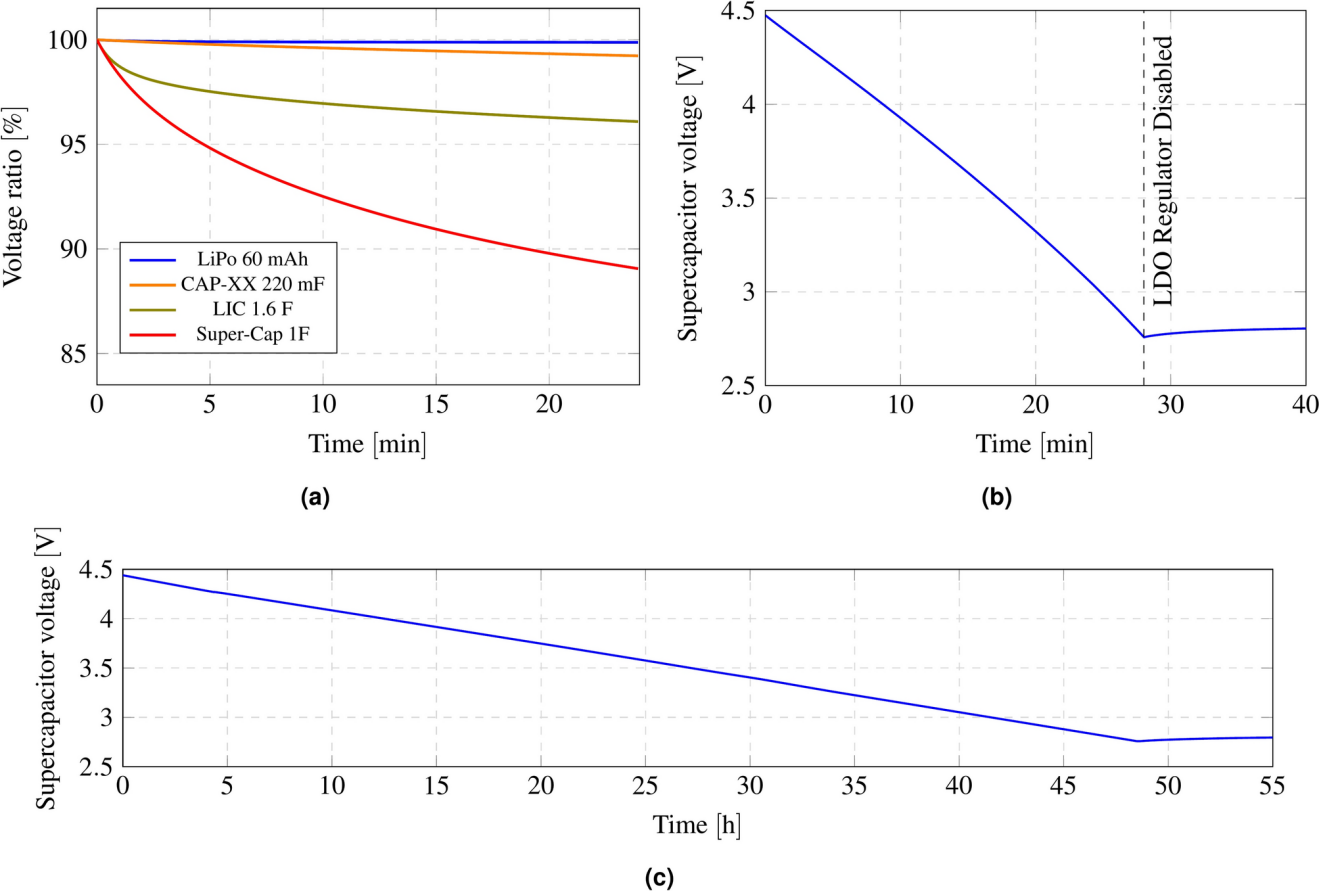
Voltage ratio of several storage elements during discharge (**a**); Voltage of the supercapacitor as a function of time while the tag is in active mode (with the reflection amplifier enabled) (**b**) and in sleep mode (**c**).

To select the storage element, a comparison is made among some of the most commonly used in low-power applications: a lithium polymer battery, a standard supercapacitor, a prismatic supercapacitor from CAP-XX, and a lithium-ion capacitor. In solar energy harvesting, it is important to ensure that the storage device is able to maintain energy long enough to avoid losing power during non-light periods. Figure 10a shows the ratio between the nominal voltage and the discharged voltage, expressed as a percentage, for the four storage elements. As expected, the lithium polymer battery exhibits the lowest energy leakage. Additionally, these batteries offer higher energy density compared to supercapacitors. However, supercapacitors are known for supporting a higher number of charge/discharge cycles, translated to a considerably longer lifespan compared to lithium polymer batteries. Fortunately, newer technologies in supercapacitor’s design leverage the advantages of both worlds, such as lithium-ion supercapacitors.

For this application, a 220 mF prismatic supercapacitor from CAP-XX (DMT3N4R2U224M3DTA0) is selected, as it provides a discharge rate very close to that of the lithium polymer battery while maintaining the longer lifetime of supercapacitors. The value of the supercapacitor is calculated based on several criteria. The minimum charge to which the supercapacitor’s voltage can drop is the minimum required by the LDO regulator to maintain the 1.8 V output voltage. Thus, the supercapacitor voltage can range from 4.2 to 2.8 V $(\Delta {\rm V})$. The leakage current of the supercapacitor (3 $\upmu {\rm A}$), as well as the microcontroller’s consumption in sleep mode (<1 $\upmu {\rm A}$), are taken into account when sizing the supercapacitor. Its value must be calculated within the most restrictive case of light exposition. This factor is not constant, as it varies depending on geographical coordinates. For example, in countries like Italy or Spain, the shortest periods of daylight last about 9 h, whereas in northern regions such as Iceland, this can decrease to around 4 h. Therefore, considering the most restrictive scenario, where the system must operate for 20 h $(\Delta {\rm t})$ without an energy source, the capacitance can be calculated mathematically by means of Eq. (2) assuming a discharge current $(I_{discharge})$ of 4 $\upmu {\rm A}$, with a resultant value of 206 mF. From the CAP-XX family of capacitors, the nominal value selected is the next available option above this calculation.

2 $$\begin{aligned} C = \frac{I_{discharge}\cdot \Delta t}{\Delta V} = \frac{(4\cdot 10^{-6}\,A) \cdot (20 \,h\cdot 3600\,s/h)}{(4.2\,V - 2.8\,V)} = 206\,mF \end{aligned}$$

Figure 10b shows the voltage of the storage element (supercapacitor connected to the BATT pin) as a function of the operation time in active mode. When sensor is triggered, tag turns on the RF front-end to backscatter some LoRa packets towards the receiver before going back to sleep. Figure 10b validates the energy stored at the 220 mF supercapacitor is several orders higher than the required by the tag to trigger the alarm (less than a second in the best scenario). Figure 10c also shows the measurement of the supercapacitor voltage in the absence of an energy harvesting source, with the tag in sleep mode. The results validate that the system can remain powered for more than 20 h without light.

It can be observed that when the voltage drops to 2.8 V, the LDO regulator output of the AEM10941 is disabled until sufficient energy is harvested to turn it on again. In the highest power consumption scenario (with the reflection amplifier activated), the tag can backscatter packets continuously for 28 min. In cases where the reflection amplifier is turned off (e.g., when the tag is close to the receiver or transmitter), it can remain in active mode for more than 5 h. Considering that the time-on-air of a single LoRa packet is 663.53 ms for the settled configuration (SF = 12, BW = 125 kHz, preamble length = 8, coding rate (CR) = 1, and CRC disabled), when the sensor is triggered, it can send 2533 packets with the reflection amplifier active and 29683 packets with the reflection amplifier disabled before running out of battery. In other words, accounting for a safety factor of 10, each time the sensor is triggered, the backscattering front-end will be active long enough to send 10 packets. Therefore, each sensor could be triggered 253 times with the reflection amplifier enabled and 2968 times with it disabled before exhausting the supercapacitor charge.

#### Control unit

**Fig. 11:**
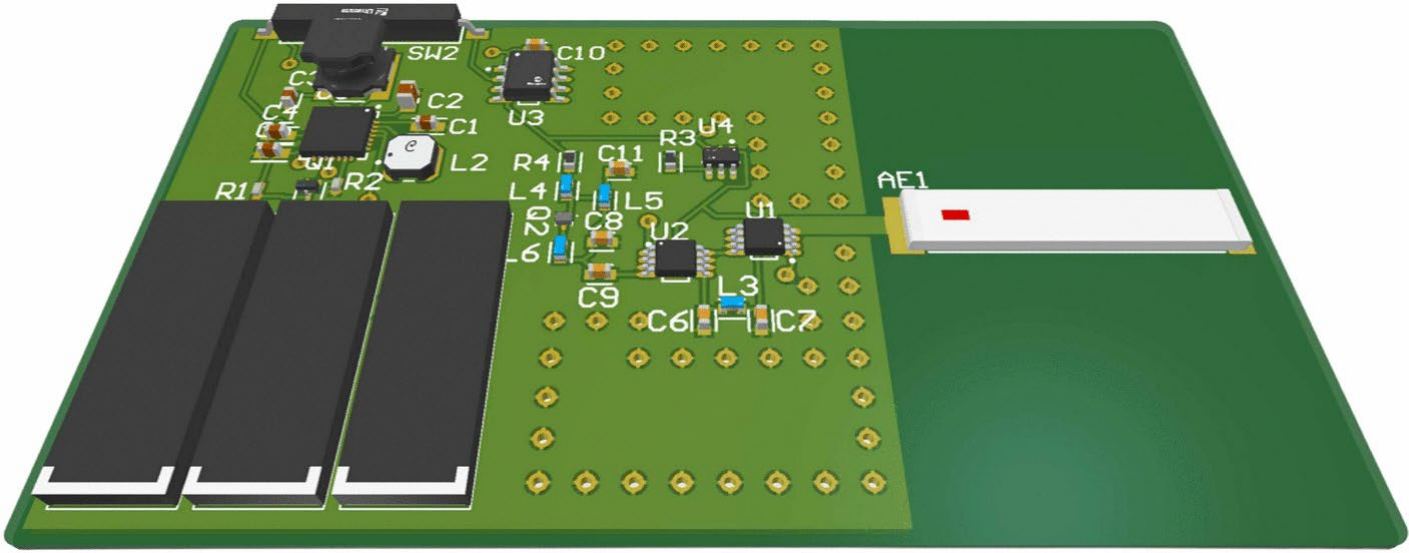
Render of the printed circuit integrating the four parts described in this section: reflection amplifier, phase modulator, control unit, and energy harvesting unit. The 3D render was generated using Altium Designer.

The control unit is responsible for activating the radio frequency front-end when the sensor is triggered. To achieve this, a low-power Attiny402 microcontroller from Microchip is utilized. The sensor connects to one of the I/O pins of the microcontroller, which remains in sleep mode most of the time and is activated by an interrupt when the sensor is triggered. Upon entering IDLE mode, the microcontroller powers the reflection amplifier, phase modulator, and oscillator, which generates the tag’s modulation signal. This signal can be generated in two ways: using the microcontroller’s internal PWM or via an external oscillator. The first option is less costly, requiring only the microcontroller; however, it is less efficient in terms of power consumption, as generating a signal of 300 $\pm \Delta {\rm f}$ kHz necessitates using the 1 MHz clock along with a prescaler, which increases the microcontroller’s consumption. The second option requires an additional external oscillator but enables the microcontroller to operate with a 32 kHz clock. Despite the combined consumption of the microcontroller at 32 kHz (7 $\upmu {\rm A}$) and the external oscillator (26 $\upmu {\rm A}$), the overall power consumption is still lower than that of the microcontroller generating the signal with PWM (455 $\upmu {\rm A}$). Therefore, the second option is selected, utilizing an LTC6906 oscillator from Analog Devices. Table 2 presents the current consumption of the polarized tag at 1.8 V, broken down by components. Table 3 details the cost of each component of the tag. The listed prices correspond to orders of at least 50 units; however, for large-scale production, costs would be lower. The total cost of the tag is estimated to be around 20 euros. Additional costs, such as PCB manufacturing, capacitors, and inductors, may slightly increase or decrease the final price, depending on the supplier, the order quantity, and the PCB surface finish. Figure 11 provides a rendered illustration of the four components described above, integrated with a 433 MHz SMD ceramic antenna.

**Table 2:** Breakdown of tag power consumption.

Part	IC	Current [$\mu {\rm A}$]	Power [$\mu {\rm W}$]
Reflection amplifier	BFR340F	322.9	581.22
Phase modulator	ADG918	$\sim$2	$\sim$3.6
Oscillator	LTC6906	21	37.8
Microcontroller	AtTiny402	7	12.6

**Table 3:** Breakdown of tag component costs.

Section	Component	Cost/unit [€]
RF front-end	BFR340F	0.129
	RF Switch (ADG918)	2.93
	Oscilator (LTC6906)	2.38
	Ceramic antena (0433AT62A0020E)	1.46
Energy harvesting	Energy Managment IC (AEM10941)	2.55
	Monocrystalline Solar Cell	2.16
Control unit	Microcontroller (AtTiny402)	0.447

### On-site measurements

**Fig. 12:**
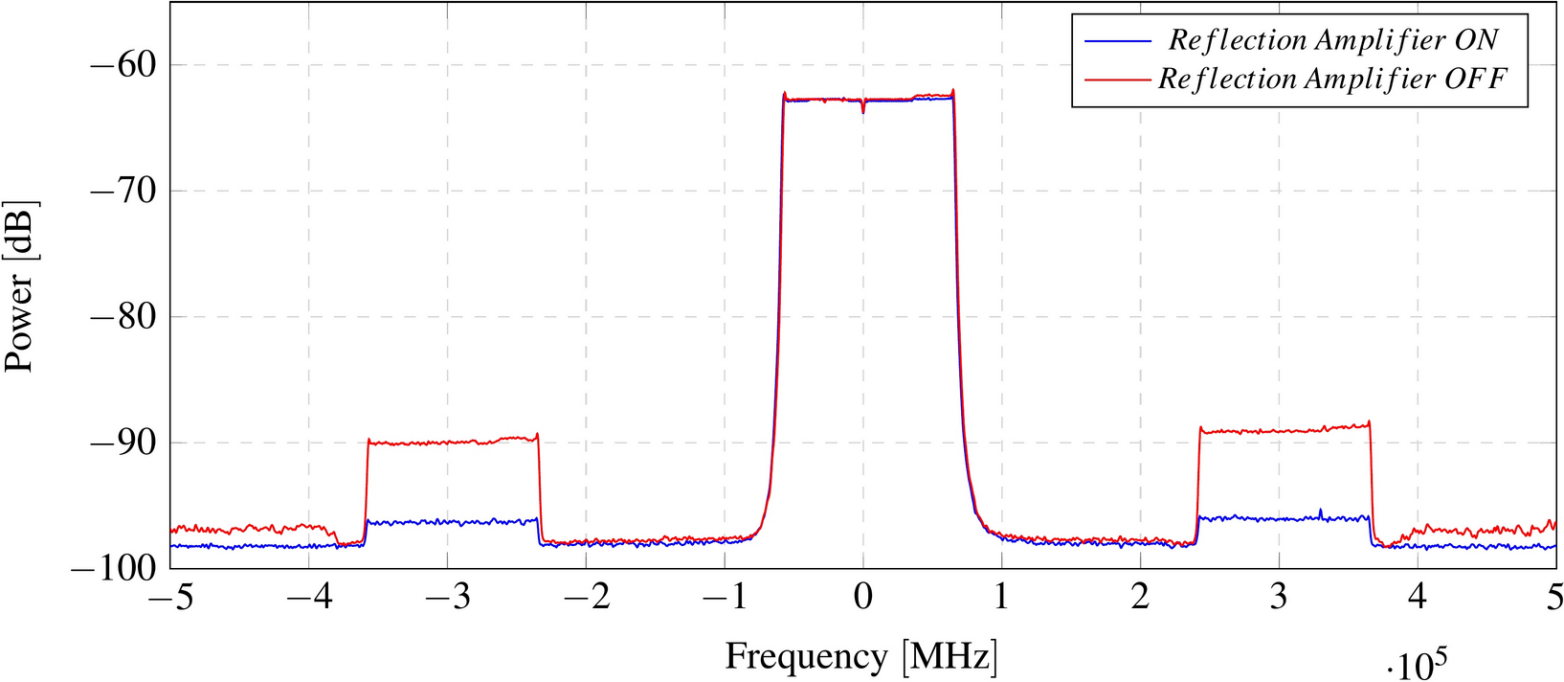
Spectrum of backscattered sideband signals when reflection amplifier is turned on (red) and off (blue).

Early experiments are carried out in the laboratory using monopole antennas at 433 MHz. Figure 12 shows a comparison of the spectrum when the reflection amplifier is turned on and off. Signals are recorded using an Adalm Pluto software-defined radio (SDR) as a receiver and a commercial LoRa transceiver as a transmitter. The transmitter and receiver are positioned at distances of 3 m and 0.5 m from the tag, respectively. LoRa is configured with a spreading factor of 12 and a bandwidth of 125 kHz

**Fig. 13:**
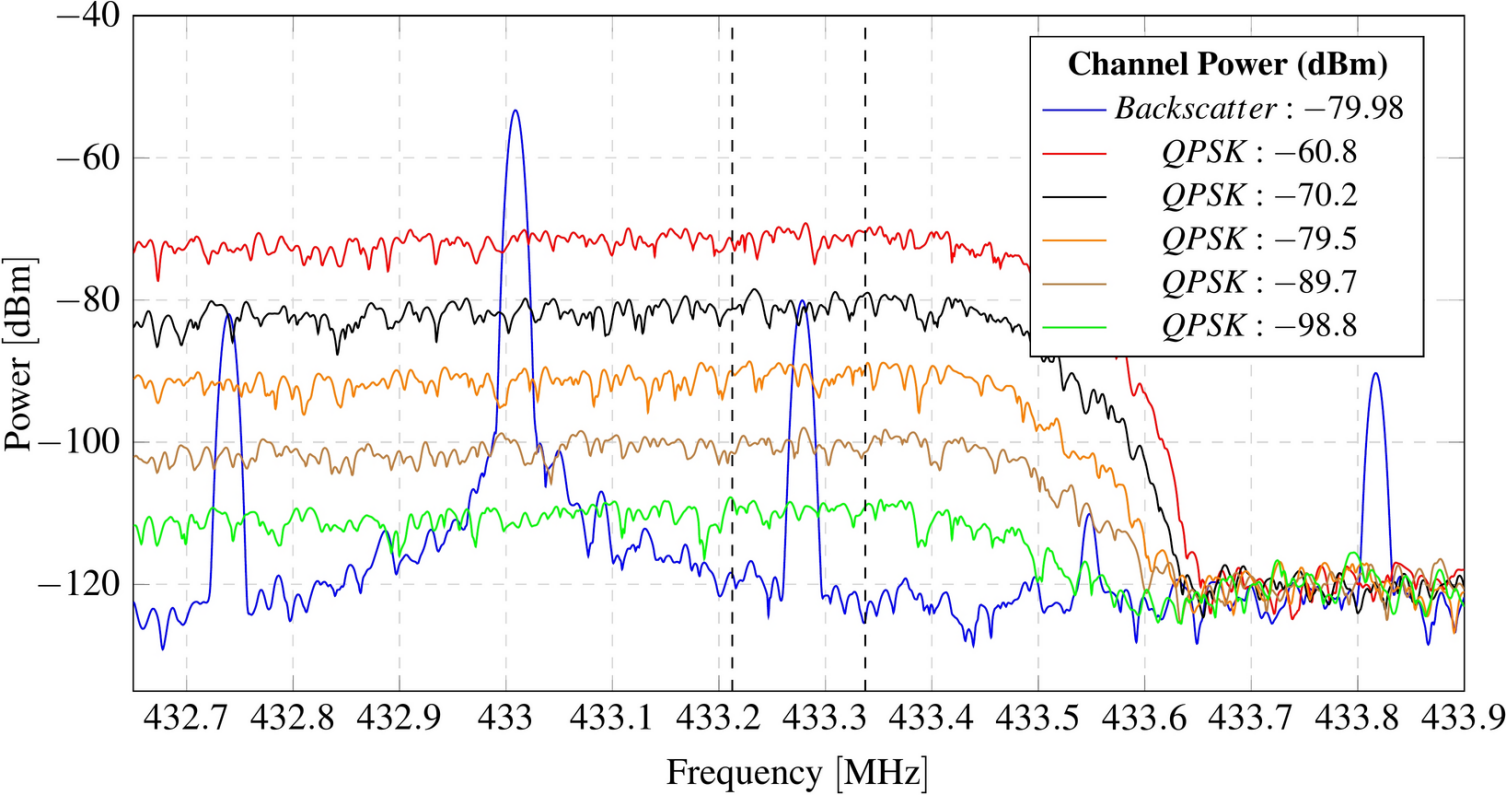
Spectrum of backscattered sideband signal over diferent levels or interference (QPSK signal).

**Fig. 14:**
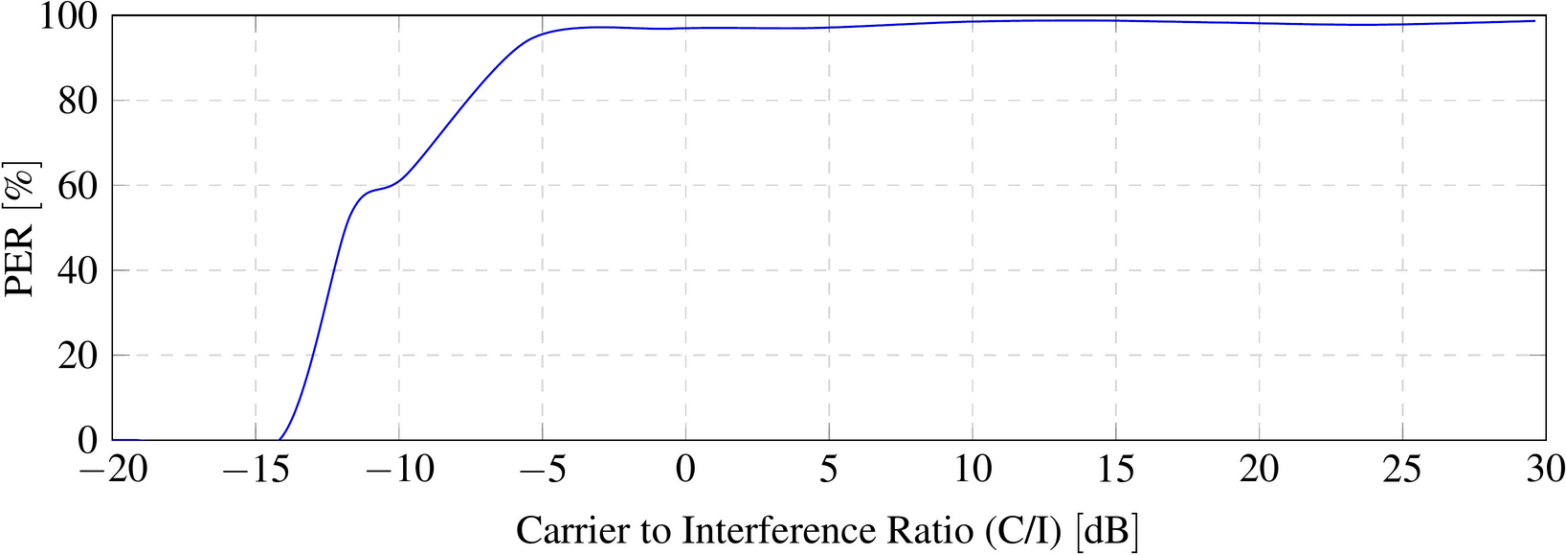
Packet error rate (PER) as a function of carrier-to-interference (C/I) rate.

To assess the system’s resilience to interference, a broadband signal capable of interfering both backscattered sidebands, is generated. A QPSK signal with a bandwidth of 1.3 MHz is transmitted with the signal generator, and its power level is progressively increased. The interference power is measured close to the receiver antenna using a spectrum analyser connected to an antenna identical to that of the receiver, to ensure consistency of the measurements. The spectral power of both the backscattered LoRa signal and the interference is evaluated within the 125 kHz-wide sideband channel. Figure 13 presents the spectrum of the backscattered LoRa signal along with the QPSK interference at diferent power levels. As anticipated, the receiver successfully demodulates LoRa packets even under negative carrier-to-interference (C/I) ratios, owing to the processing gain inherent to CSS modulations, which enables demodulation of signals below the noise floor. Figure 14 illustrates the packet error rate (PER) as a function of the C/I ratio. For C/I ratios equal to or greater than 0 dB, the PER remains below 3%. However, as the C/I ratio drops below 0 dB, the packet loss rate starts to increase rapidly. Specifically, for C/I ratios of − 9.78 dB and − 11.68 dB, the PER reaches 37.74% and 46.75% of lost packets, respectively.

**Fig. 15:**
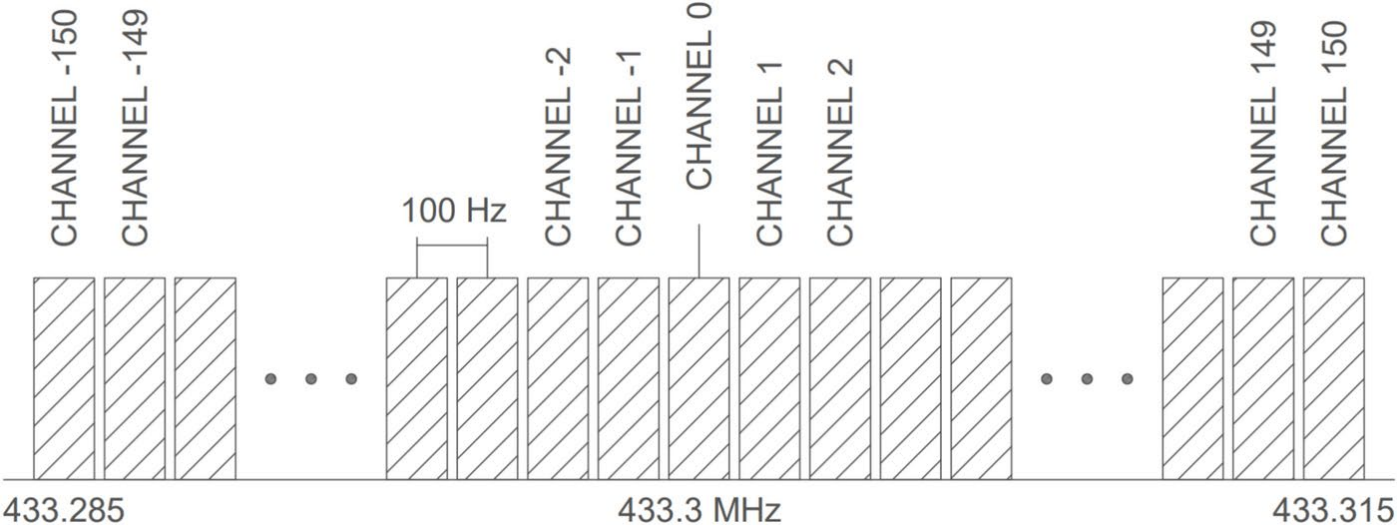
Diagram of the channel configuration used in the experimental measurements.

To validate the accuracy of determining the provenance of a received packet (by checking the frequency deviation from the central frequency), a backscattering tag is modulated at 301 different frequencies. Each modulation represents one possible channel, with channels spaced 100 Hz apart. The channel separation of 100 Hz is set to be four times greater than the frequency error of the TCXO ($\pm 26 {\rm Hz}$), as determined in the Tag Identification subsection. LoRa can demodulate packets with a frequency deviation of $\pm 31.25$ kHz; however, the experiment is conducted within half of that range ($\pm 15$ kHz) to avoid operating at the theoretical limit. The results indicate that the frequency deviation detected by LoRa commercial transceivers is influenced not only by the oscillator’s frequency drift but also by the magnitude of the frequency deviation relative to the central channel. Figure 15 shows a diagram of the channels evaluated experimentally. In this application, each wireless sensor in the system is configured to transmit on a single, unique channel. Therefore, accurately identifying the channel is crucial for determining which tag is transmitting.

**Fig. 16:**
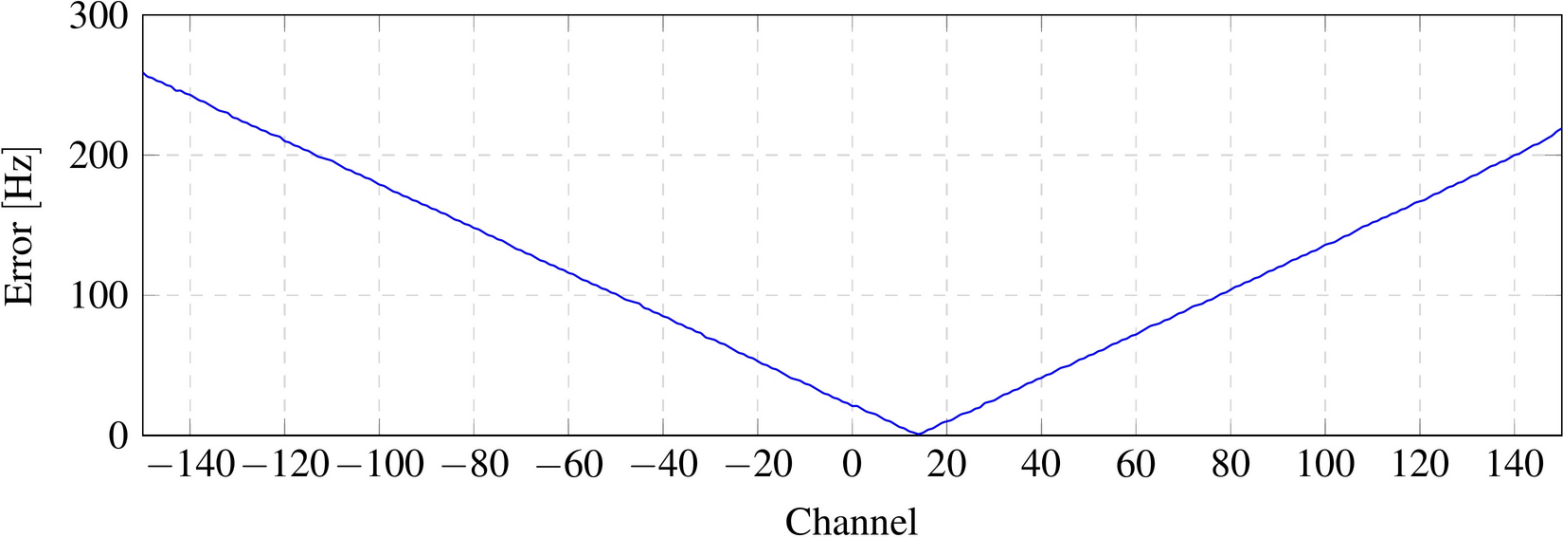
Frequency deviation from the tag modulation frequency (– 15 to 15 kHz) at each channel.

**Fig. 17:**
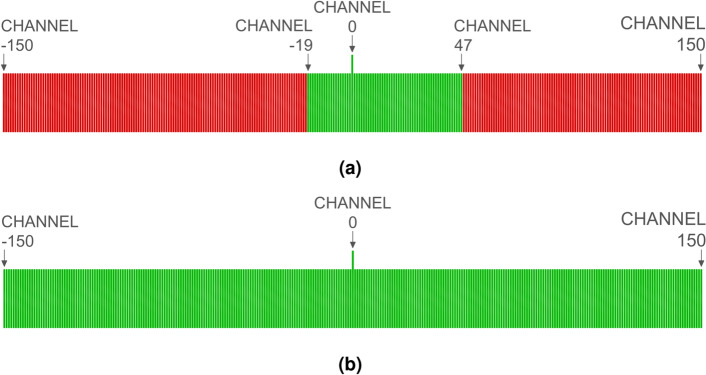
Channel correct/incorrect determination before (**a**) and after (**b**) compensating for the frequency deviation introduced by the LoRa receiver.

Figure 16 illustrates the frequency error at each channel, showing an increase in error as the frequency moves away from the central frequency (channel 0 at 300 kHz). Figure 17 shows the channel determined by the receiver before and after compensating for the frequency error introduced by the receiver.

It is important to clarify that the application proposed in this article (a wireless security system) does not need to address issues related to simultaneous communications from multiple sensors, as required by other applications. This is because an intrusion attempt typically targets a single specific entry point. In the unlikely event that access is attempted through multiple points simultaneously, the probability of triggering the sensors at the exact same moment is particularly low. Nevertheless, assuming the worst-case scenario where two or more sensors are activated at the same instant, it is sufficient to detect part of the preamble from one of the packets (presumably the one closest to the transmitter/receiver) to trigger the alarm. Therefore, the simultaneous activation and backscattering of multiple packets at the same time do not pose any issue.

Distance measurements are carried out to validate the capability of the proposed system and the range enhancement of the reflection amplifier compared to a non-amplified backscattering modulator. The measurements are carried out placing the tag 2.4 m from the transmitter and moving the receiver away in a straight line.

**Fig. 18:**
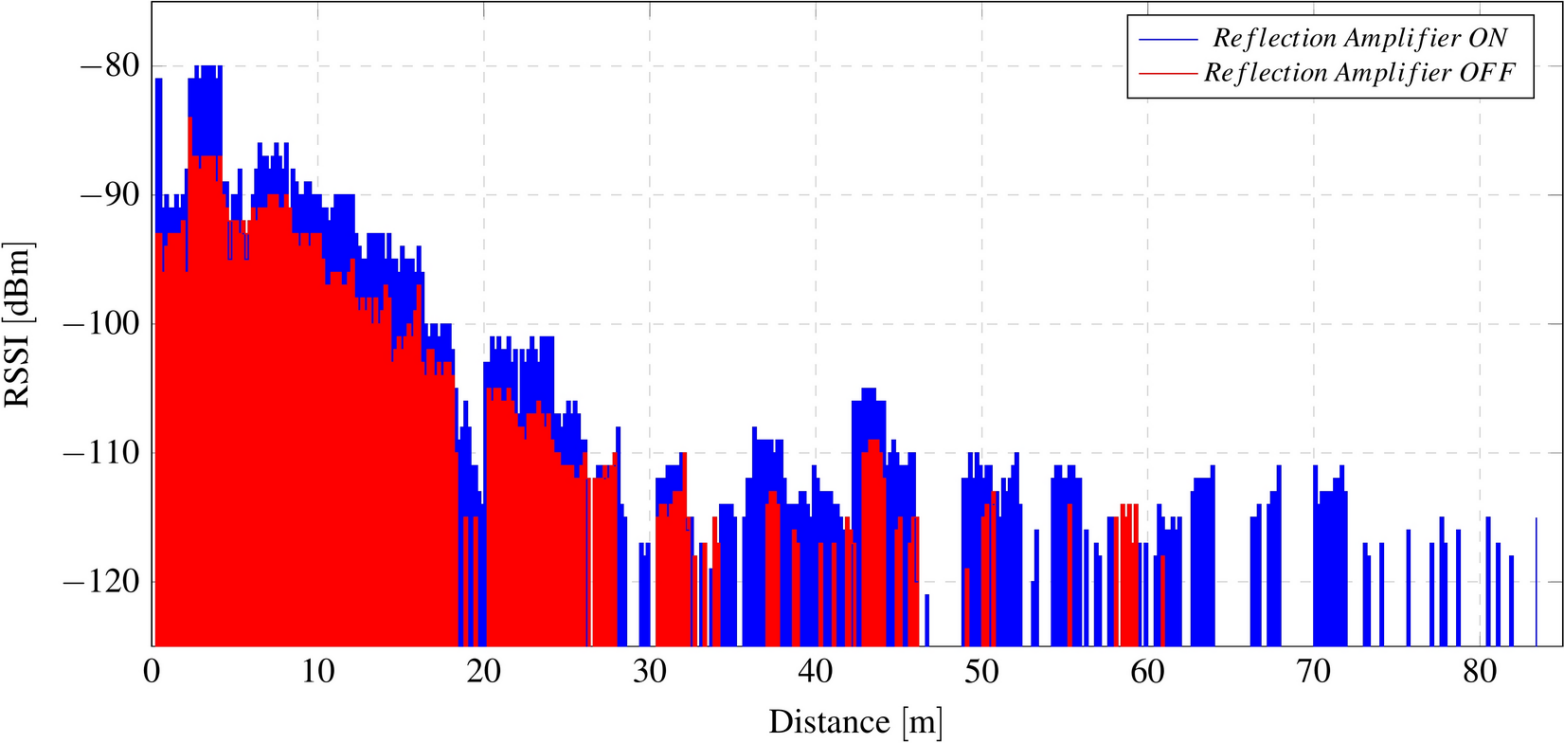
Received signal strength indicator (RSSI) as a function of distance.

The measurements are conducted in a hallway with a direct line of sight. It is important to note that this environment is not ideal for wave propagation, as waves do not travel freely without obstacles; rather, numerous interferences are caused by the floor, walls, and railing, which create intermediate points where the signal is lost^47,48^. The transmission power is set at 10 dBm. Figure 18 shows the received signal strength (RSSI) as a function of distance. A photograph of the measurement scenario is provided in the Methods section.

**Fig. 19:**
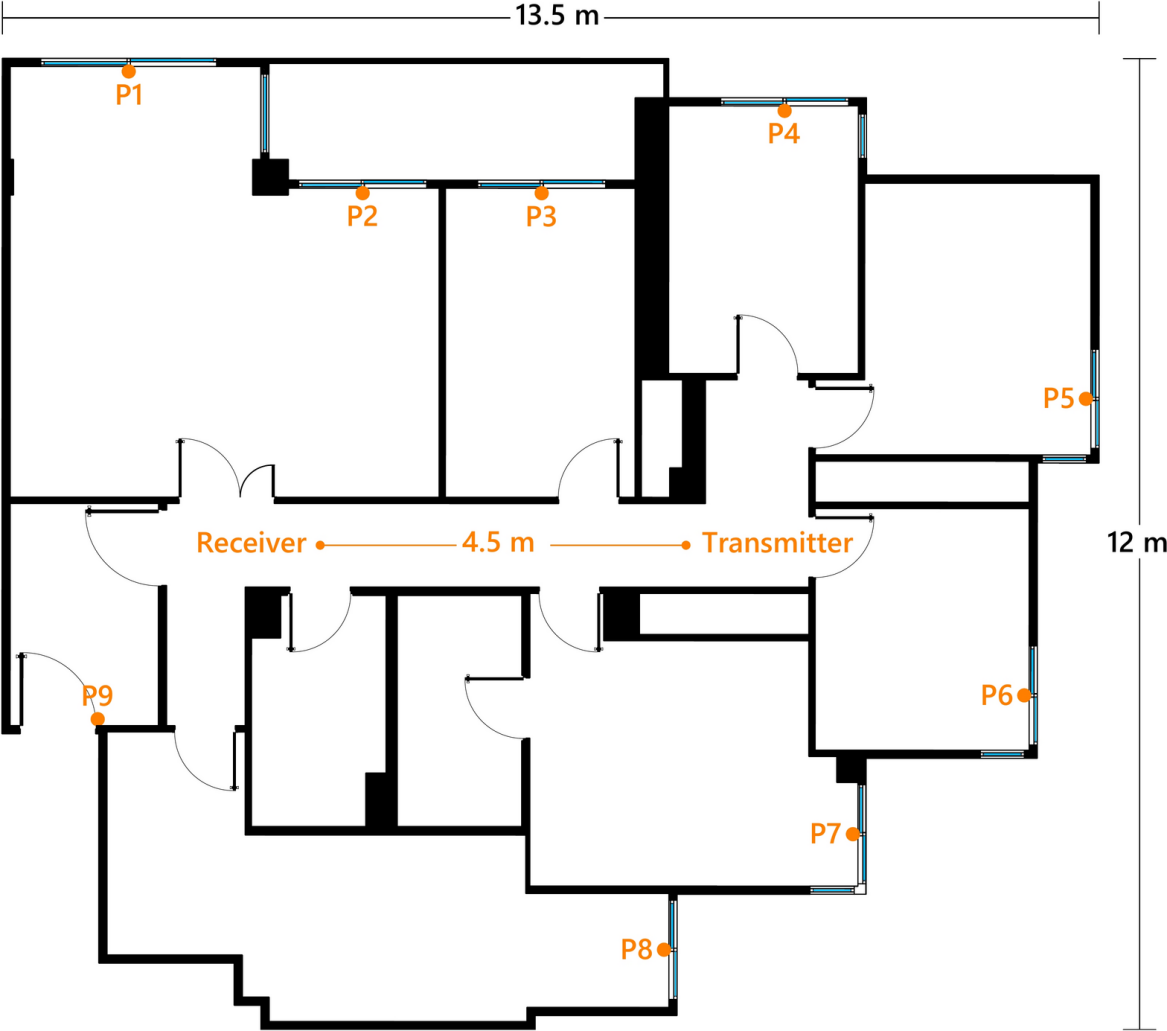
Layout of the apartment where measurements are conducted, showing the positions of the transceivers and the assessment points for the wireless sensor (P1–P9).

Finally, the system is validated in a real-case scenario, demonstrating its capability to cover a large household (150 $m^2$) with a single transmitter and receiver, as shown in Fig. 19. LoRa transceivers are placed 4.5 m apart, and critical points for potential external access (main entrance and windows) are assessed by placing the wireless sensor at these locations. A total of nine points (P1–P9), illustrated in Fig. 19, are tested.

The system is evaluated at two transmission power levels (10 dBm and 0 dBm) with the reflection amplifier both enabled and disabled, measuring the RSSI and SNR of the received packets. The measurement results are provided in Table 4. From the table, it can be observed that P4 and P5 are the most critical points despite not being the furthest from the transmitter. This measurement demonstrates that, even when the distance is within the system’s maximum coverage range, environmental factors do not guarantee sensor coverage. Furthermore, the measurements show how the reflection amplifier resolves the coverage failure at points P4, P5, and P8. It can also be noted from the table’s results that the reflection amplifier is more effective when the transmission power is lower or when the wireless nodes are farther from the transmitter. This behavior is known and supported by the amplifier’s characterization in the laboratory, where it was observed that the gain decreases as the input power increases, a consequence of the amplifier’s gain compression.

It is important to mention that the transmitter’s maximum power is 22 dBm. In Spain, as stipulated by the Spanish standardization agency (UNE) in the UNE-EN 300220-2 standard^49^, and similarly to other European countries, transmissions in the 433 ISM band cannot exceed 10 dBm. However, in other countries, these regulations may vary and can be more lenient, permitting higher power levels. Consequently, the system’s final configuration strongly depends on local regulations and the installation environment. For example, in the apartment depicted in Fig. 19, higher transmission power levels could allow for deactivating the amplifier across all wireless sensors, significantly reducing their energy consumption.

**Table 4:** Measurements of RSSI and SNR in the apartment depicted in Fig. 19.

Tx Power	Reflection Amplifier	Measure	Position								
			P1	P2	P3	P4	P5	P6	P7	P8	P9
0 dBm	Enabled	RSSI	− 100.11	− 101.09	− 90.52	− 106.8	− 115.36	− 93.62	− 98.49	− 107.57	− 98.01
	Disabled		− 116.27	− 115.12	− 101.20	−	−	− 106.67	− 107.55	−	− 110.36
	Enabled	SNR	− 2.04	− 2.56	2.58	− 7.01	− 15.69	1.96	− 0.98	− 8.11	− 0.93
	Disabled		− 16.45	− 15.16	− 2.44	–	–	− 7.08	− 7.95	–	− 10.56
10 dBm	Enabled	RSSI	− 93.56	− 92.02	− 82.9	− 101.0	− 98.15	− 88.62	− 86.52	− 96.08	− 93.0
	Disabled		− 105.53	− 101.32	− 86.69	–	− 111.53	− 99.98	− 102.09	− 105.02	− 100.23
	Enabled	SNR	− 8.19	− 14.84	1.39	− 9.17	− 5.84	− 11.85	− 0.19	− 5.72	− 2.2
	Disabled		− 12.42	− 8.02	1.15	–	− 19.6	− 7.07	− 9.15	− 11.85	− 7.25

## Methods

### Prototype manufacturing

**Fig. 20:**
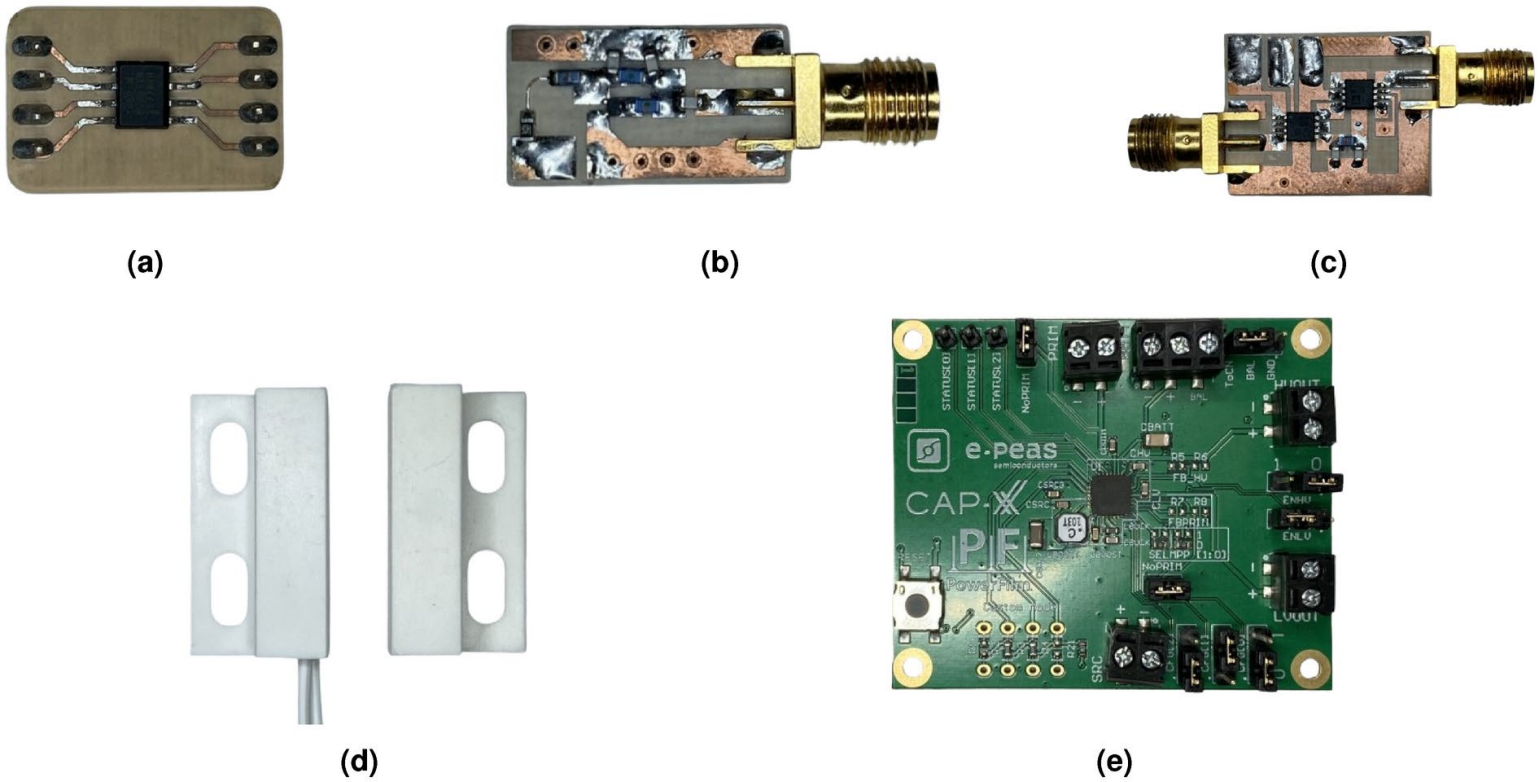
Photograph of the prototype components: (**a**) control unit (microcontroller AtTiny402); (**b**) reflection amplifier (BFR340F - Infineon Technologies); (**c**) phase modulator (ADG918 - Analog Devices); (**d**) aperture sensor; and (**e**) energy harvesting development board (AEM10941 - E-peas).

The radiofrequency backscattering front end is implemented using a Rogers 4003C substrate, a high-frequency laminate with the following technical specifications: a substrate thickness of 32 mils, a copper thickness of 34 $\mu$m, a dielectric constant of 3.55, and a dissipation factor of 0.0027. The printed circuit boards (PCBs) are manufactured using a computer numerical control (CNC) machine, which mills the outline of the copper paths. Subsequently, unused copper is removed by means of the peeling technique. To ensure proper shielding, RF lines are enclosed using 0.8 mm riveting vias. The reflection amplifier and modulator are interconnected with an SMA connector. Figure 20 illustrates the different components of the prototype.

### Measurement setup

A comparison of the frequency deviation between the oscillators of the two main families of LoRa transceivers is conducted using two low-cost development boards. For the SX127x transceiver family, a TTGO LORA32 board is utilized, while a Heltec wireless stick V3 board is employed for the SX1262 family. Both boards are configured for the 433 MHz band. The measurements are taken with the receiver placed indoors at room temperature, while the transmitter, equipped with a temperature sensor, is positioned outdoors for a duration of two days. This setup enables the measurement of fluctuations in the central frequency of the channel as a function of temperature.

Frequency deviation measurements are conducted in the laboratory by controlling the modulation frequency of the tag using a RIGOL DG1062Z function generator. Interference measurements are performed in the laboratory by generating a QPSK signal and progressively increasing its power level. The QPSK bandwidth has been set to 1.3 MHz. The power of both the backscattered signal and the interference is assessed by placing a FPC1500 spectrum analyzer, from Rohde and Schwartz, near the receiver’s antenna. While increasing the interference power, the number of received packets is recorded from the receiver’s serial monitor to subsequently calculate the packet error rate (PER).

**Fig. 21:**
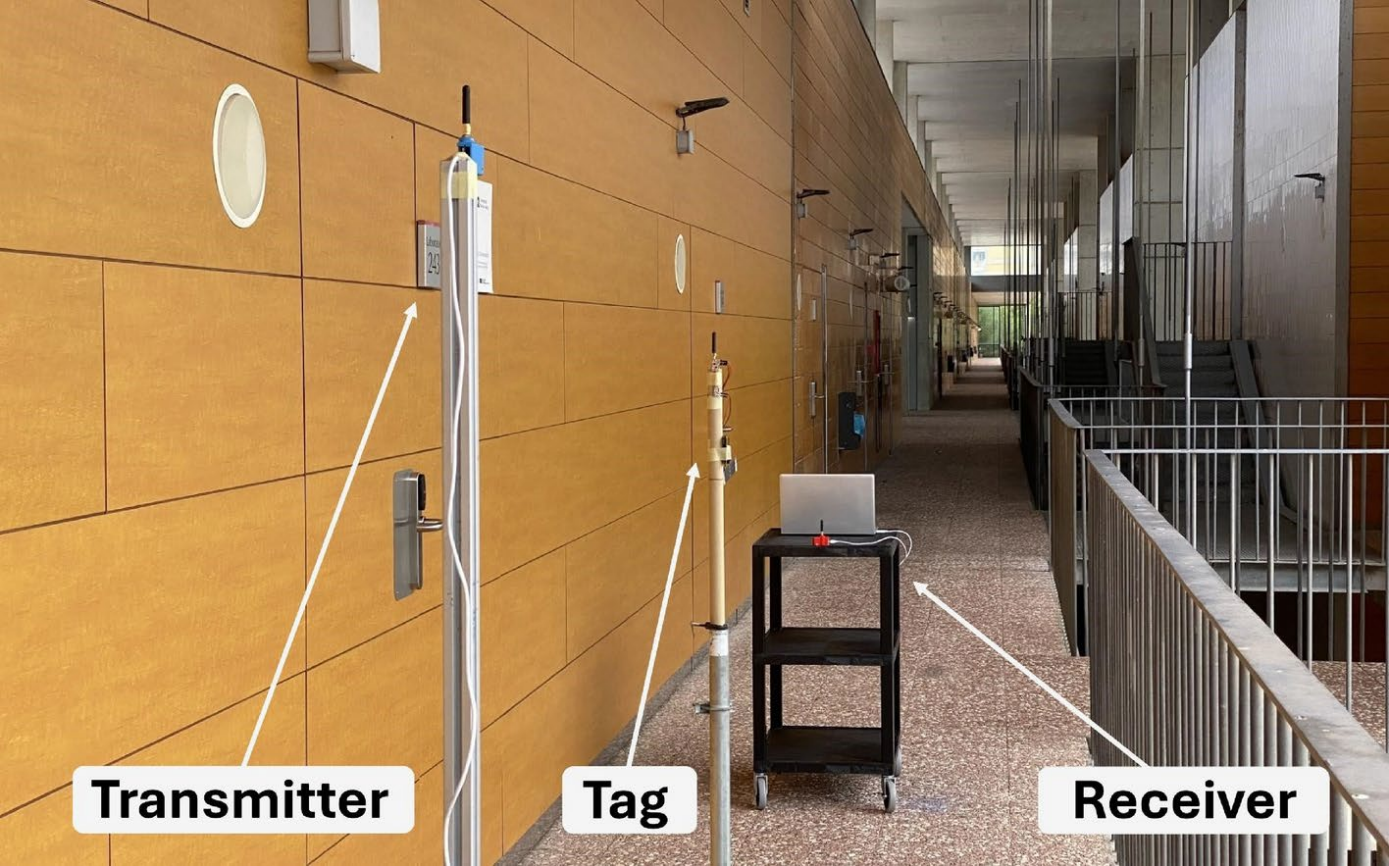
Photograph of the measurement setup and environment used to validate the coverage enhancement of the reflection amplifier.

Coverage measurements, as shown in Fig. 18 from the previous section, are conducted in a hallway with a direct line of sight. The transmitter and tag are positioned 2.4 m apart, while the receiver is gradually moved farther away until the signal is completely lost. The transmitter power is fixed at 10 dBm. All measurements presented in this paper were performed using the quarter-wave monopole antennas that come standard with the LoRa modules, which typically have a gain of 2 dBi. Figure 21 provides a photograph of the measurement environment, illustrating the positions of the transmitter, tag, and receiver.

## Conclusion

This work presents a surveillance system based on LoRa backscattering that improves several aspects of existing commercial systems. First, the power consumption of the wireless nodes is reduced by replacing conventional front ends with a backscattering-based front end. This reduction in power consumption allows the use of a supercapacitor recharged by solar energy instead of typical button or LiPo batteries. Additionally, the ASK modulation used in commercial systems is replaced with LoRa modulation, making the system more resistant to interference and increasing the range of the backscattered signal. Second, a reflection amplifier with a phase modulator is designed and integrated in the backscattering front end to increase de reflected power, and consecuently enhance the range. To identify which sensor triggers the alarm, a novel detection protocol is proposed based on the inherent ability of LoRa transceivers to determine the frequency deviation. The modulation is entirely analog, ensuring maximum efficiency and responsiveness compared to other digital solutions. The reliability of this communication protocol is experimentally validated by backscattering LoRa packets across 301 different channels, each separated by 100 Hz.

Finally, the system is validated in indoor environments, including both laboratory settings and a real-world scenario. Low-cost commercial LoRa transceivers are employed as a cost-effective alternative to the expensive SDR equipment frequently cited in the literature. Measurements in the laboratory, conducted in a corridor with considerable multipath interference, demonstrate the range enhancement achieved by integrating the reflection amplifier, achieving a maximum of 84 meters with the reflection amplifier enabled, compared to 60 m when it is disabled. The system is also tested in an apartment with two different transmission power levels, recording the RSSI and SNR metrics of the received packets. These results highlight the importance of the amplifier in providing coverage to the most challenging points and validate the system’s operation in a real application. None of the measurements from the laboratory or the apartment exceeded a transmission power of 10 dBm. It can be concluded that the range in other scenarios can be further enhanced by increasing transmission power (when permitted by regulations), deploying additional LoRa transceivers, strategically positioning them to mitigate fading caused by multipath interference, or adjusting the CSS modulation parameters.

## Data Availability

The data are available upon request to the corresponding author.
